# In Situ Gelling Dexamethasone Oromucosal Formulation: Physical Characteristics Influencing Drug Delivery

**DOI:** 10.3390/gels11010026

**Published:** 2025-01-02

**Authors:** Daniel Krchňák, Ľudmila Balážová, Michal Hanko, Dominika Žigrayová, Miroslava Špaglová

**Affiliations:** 1 Department of Galenic Pharmacy, Faculty of Pharmacy, Comenius University Bratislava, Odbojárov 10, SK-832 32 Bratislava, Slovakia; krchnak6@fpharm.uniba.sk (D.K.); zigrayova1@uniba.sk (D.Ž.); 2 Department of Pharmaceutical Technology, Pharmacognosy and Botany, University of Veterinary Medicine and Pharmacy in Košice, Komenského 73, SK-041 81 Kosice, Slovakia; ludmila.balazova@uvlf.sk; 3 Department of Pharmaceutical Analysis and Nuclear Pharmacy, Faculty of Pharmacy, Comenius University Bratislava, Odbojárov 10, SK-832 32 Bratislava, Slovakia

**Keywords:** dexamethasone, aphthous stomatitis, mucoadhesion, mint essential oil, cellulose derivatives

## Abstract

The study focuses on the development of an in situ gelling dexamethasone (DEX) oromucosal formulation designed for the treatment of aphthous stomatitis. Three series of formulations were prepared; a first series containing DEX suspended, a second series containing DEX and, in addition, mint essential oil (EO), and a third series containing EO and DEX solubilized in propylene glycol (PG). In the composition, polymers in the role of mucoadhesive agent were interchanged (hydroxypropyl methylcellulose (HPMC), hydroxypropyl cellulose (HPC), hydroxyethyl cellulose (HEC), methyl cellulose (MC), carboxymethyl cellulose (CMC), and sodium carboxymethyl cellulose (NaCMC). Specifically, DEX was incorporated at a concentration of 0.1% (*w*/*w*) in each formulation. The influence of mint EO and DEX solubilization on the physical properties (pH measurements, rheological analysis, swelling ability, and texture analysis) and in vitro drug release was studied. Key findings revealed that HPMC-based formulation containing mint EO and PG exhibited best swelling properties (700 ± 46% after 5 h), adequate adhesiveness and in vitro drug release (34.7 ± 5.9%). Furthermore, the irritation potential assessed via the hen’s egg test on the chorioallantoic membrane (HET-CAM) demonstrated low irritancy risk. Finally, Fourier-transform infrared spectroscopy (FT-IR) showed no incompatibility between DEX and excipients. Overall, the research highlights the potential of mucoadhesive systems in improving the therapeutic efficacy of oromucosal drug delivery for managing painful oral lesions.

## 1. Introduction

Aphthous stomatitis, commonly known as canker sores, is a prevalent condition characterized by painful ulcerations in the oral mucosa [1]. Although not life-threatening, these lesions can significantly impair the quality of life due to associated symptoms such as pain, inflammation, and difficulty in eating or speaking [2].

The precise etiology of aphthous stomatitis remains elusive, complicating effective management strategies. Current therapeutic approaches primarily focus on symptomatic relief, often utilizing conventional dosage forms that have limited retention on the moist oral mucosa [3]. This results in suboptimal bioavailability and transient therapeutic effects, necessitating the exploration of innovative drug delivery systems. Recent advancements in polymeric mucoadhesive systems present a promising solution to the limitations of traditional treatments [4]. These systems are designed to adhere to the mucosal surface, forming a gel-like structure that can prolong the retention of therapeutic agents at the site of action due to the presence of mucoadhesive polymer. Examples of such excipients include carbomers, chitosan, sodium carboxymethyl cellulose, hydroxypropyl methylcellulose, hydroxypropyl cellulose, and methyl cellulose. Therefore, these were tested and varied, in the proposed formulations. The principle of mucoadhesion involves two stages: the contact stage and the consolidation stage. During the contact stage, the mucoadhesive formulation comes into the contact with the mucus membrane, causing the polymer to spread and swell, resulting in deep contact with the mucus layer. The consolidation stage occurs when moisture activates the mucoadhesive polymer(s). The presence of moisture allows the mucoadhesive molecules to break free and form weak van der Waals and hydrogen bonds [5].

Oromucosal drug delivery systems used to treat aphthous stomatitis include liquid, semi-solid, and solid medicines. Among solid-dosage forms, tablets, wafers, films, and patches are the most common [6]. Gels are particularly easy to prepare. Recent studies indicate that these gels often incorporate drugs embedded in nano-carrier systems. For instance, Karavana et al. [7] developed bioadhesive gels containing cyclosporin A within solid lipid nanoparticles. Farsi et al. [8] incorporated DEX into liposomes that were subsequently dispersed in the gel. The authors referred to this system as gelosome. The rationale for using liposomes was to mitigate potential systemic effects and to prolong drug release. In addition to mucoadhesive gels, mucoadhesive films also show great promise. Arafa et al. [9] formulated niosome-based mucoadhesive films containing propolis. Finally, a fast-dissolving oral thin film containing DEX was developed by Shimoda et al. [10], although primarily for a different therapeutic purpose, namely for antiemesis during chemotherapy.

DEX as a synthetic glucocorticoid with anti-inflammatory and immunosuppressive properties [11] belongs to the first choice of steroidal anti-inflammatory drug prescribed by dentists [12]. The mechanism of action of DEX involves its binding to the glucocorticoid receptor (GR), which leads to the modulation of gene expression. Upon binding, the DEX-GR complex translocates to the nucleus and interacts with glucocorticoid response elements in DNA [13]. This results in the upregulation of anti-inflammatory proteins and the downregulation of pro-inflammatory mediators, effectively inhibiting leukocyte infiltration at sites of inflammation [14], suppressing the function of various immune mediators, and reducing pain and swelling [15]. In the context of oromucosal therapy, DEX can be administered through the buccal mucosa, providing a localized effect while minimizing systemic exposure [16].

Peppermint essential oil, typically obtained through the steam distillation of aerial parts of the flowering plant—Mentha piperita, with following redistillation or rectification to remove sulfur compounds [17]—is recognized for various therapeutic properties, including its role as a penetration enhancer in pharmaceutical formulations [18]. Its inclusion in drug delivery systems, particularly for oromucosal applications, has gained attention due to several beneficial characteristics, including its ability to enhance permeability across mucosal membranes [19], provide a cooling sensation that can mask unpleasant taste or pain [20,21], and potentially exhibit antimicrobial properties that could prevent oral infections [22]. Peppermint oil’s unique combination of menthol, menthone, and other terpenes [17] is thought to contribute to these desirable effects.

The pharmacological management of aphthous stomatitis presents several challenges. One such challenge is the humid environment of the mouth, which can cause the medication applied to the affected area to dissolve in saliva. This results in undesirable systemic effects and necessitates frequent reapplication of the treatment. Traditional therapies for aphthous ulcers include corticosteroids, local antiseptics, and local anesthetics [23]. There are several mucoadhesive pastes available on the market for this condition, such as Turbocort, Kenacort, Kenalog, and Orabase paste with hyaluronic acid. However, in our country, there is still an interest in individually prepared medications. This interest has motivated our experimental exploration in this area.

The study aimed to develop an optimal in situ gelling DEX mucoadhesive formulation for the treatment of aphthous stomatitis, based on the known composition of the formulation presented in the study as A1 (reference sample) [24]. This involved investigating various cellulose derivatives—hydroxypropyl methylcellulose (HPMC), hydroxypropyl cellulose (HPC), hydroxyethyl cellulose (HEC), methyl cellulose (MC), carboxymethyl cellulose (CMC), sodium carboxymethyl cellulose (NaCMC) and other excipients, to enhance drug delivery. A suspended polymer in a lipophilic basis forms an in situ gel in the moist oral cavity, which can retain the product on the lesion for several hours. The study determined the effect of various cellulose derivatives in the formulation on mucoadhesion. Additionally, the influence of other excipients, such as peppermint essential oil and propylene glycol (PG), on in vitro drug delivery was investigated.

## 2. Results and Discussion

Aphthous stomatitis is a non-life-threatening condition causing discomfort in the oral cavity due to lesions resulting from mucosal epithelium necrosis. The etiology of this condition is not clear. Therefore, the therapy is focused on providing symptomatic treatment. The oral cavity’s moist environment makes it challenging to maintain conventional dosage forms on the mucosa for an extended period, leading to low bioavailability and short-term effectiveness of drugs. In situ forming gels, with strong mucoadhesive properties, offer a solution to this problem. This study compares the efficacy of a reference mucoadhesive formulation containing 0.1% (*w*/*w*) DEX [24] with the others differing in the content of the mucoadhesive polymer, the presence of peppermint essential oil, and the incorporation of DEX in PG solution. The reference formulation is widely used in dentistry. DEX, a prescription steroidal anti-inflammatory drug, is used off-label in this case, to reduce inflammation in recurrent aphthous stomatitis.

Cellulose derivatives suspended in a lipophilic base form a gel structure in the moist environment of the oral cavity with excellent mucoadhesiveness, capable of retaining the preparation on the lesion for several hours. The reference polymer HPMC was replaced with one of the following—MC, HEC, NaCMC, CMC, or HPC. Apart from studying the impact of polymer substitution on the mucoadhesion and other physical characteristics of the semisolids, the inclusion of peppermint essential oil (in the formulations A2-F2) and PG (in the formulations A3-F3) affecting the release of DEX from the in situ gels was investigated.

### 2.1. Measurement of the Actual Acidity

The pH values of the formulations ranged from 3.98 ± 0.05 to 6.45 ± 0.05 (see Table 1). The formulations C1, C2, and C3 containing HEC as the main polymer base, had the lowest values. The topical administration of these formulations may result in mild irritation of pathologically modified oral mucosal tissue, but it does not pose a serious risk. We validated this assumption through a HET-CAM test on CAM. Our results indicated that a decrease in pH resulted in increased permeability of the vascular walls, which was evidenced by the occurrence of hemorrhage. The other formulations ranged from 5.86 to 6.45. The optimal pH of oromucosal formulations may vary, depending on the specific drug administered and its pharmacokinetics. He and Mu [25] refer to the fact that an unstimulated human saliva has a pH value ranged from 6.2 to 7.6, while the average salivary pH is 6.8. Thus, the examined formulations approximately correspond to physiological values. On the other hand, a lower pH may have a positive effect on the absorption of DEX [11].

The lower pH of HEC formulations is notable, compared to other formulations. We believe this phenomenon is due to the presence of hydroxyethyl groups, which can influence the diffusion of ions and protons. This alteration may lead to interactions with other components of the formulation, especially if any of those components are acidic. While DEX itself is not classified as an acidic compound, its behavior in solution can display some acidic characteristics. This was confirmed by a more significant decrease in pH when using PG for the solubilization of DEX.

### 2.2. Rheological Behavior

The rheological properties characterization helps to determine the flow behavior and consistency of the formulation, which are critical for patient compliance and simplicity of administration in oromucosal drug delivery. As shown in Table 1, essential oils can significantly reduce viscosity, which may affect the release profile of DEX. The ΔȠ values indicate how much the viscosity changes under stress conditions. Positive values indicate that the system is dilating, due to mechanical stress. For example, pastes become harder to spread, the longer they are mixed. Formulations with rheopectic flow (i.e., two curves visible on the Figure S1) include A1, A2, A3, B1, C1, C2, E1, and F1. An interesting finding is that formulations in which DEX was incorporated as a solid drug (numerically designated as series one) behave as pastes, with formulation D1 as an exception. Conversely, the gels exhibit time-independent pseudoplastic flow or time-dependent thixotropic flow. In any case, unlike pastes, their viscosity decreases, due to mechanical stress. The case for the formulations B2, B3, C3, D1, E2, E3, F2, and F3, which show a negative value for the change in viscosity and behave as thixotropic systems, is similar. The drug is more stable in a more viscous system. However, for the drug’s bioavailability, it is advantageous if the viscosity is lower due to mechanical stress, allowing the drug to be more easily released from the system. Formulations F2 and F3 show high viscosity Ƞ_1_ and at the same time a large negative viscosity change (-), making them the most promising of the studied systems in terms of rheological properties, drug stability, and drug bioavailability. Moreover, these formulations exhibit any adverse effects on chicken CAM, which serves as a model for mucosal membranes. In summary, the rheological behavior of the formulations is significantly influenced by the type of cellulose derivative used in the composition, the presence of the essential oil, and the way DEX is incorporated. The formulations containing HPMC (A) and HPC (E) provide desirable viscosity profiles for pharmaceutical applications. The viscosities of formulations containing CMC or NaCMC (D and E) were generally low, indicating that these formulations may not be suitable for oromucosal administration requiring high viscosity because, after further dilution with saliva, they can be washed away from the site of action.

Each polymer exhibits distinct viscosity characteristics, which in turn affect its performance in a formulated oromucosal drug delivery system. For instance, HPMC demonstrated superior swelling and mucoadhesive properties, which can be directly attributed to its ability to form a cohesive and stable gel network with higher viscosity compared to other polymers like NaCMC or HPC. This higher viscosity contributes to enhanced gel stability, prolonged retention on the mucosal surface, and controlled drug release. Conversely, polymers with lower viscosities, such as NaCMC, were observed to degrade more quickly in the hydrated state, leading to reduced performance in terms of mucoadhesion and drug release.

### 2.3. Texture Analysis

Texture analysis is a simple method enabling the evaluation of mechanical features such as hardness, elongation and flexibility, but also mucoadhesion properties of various solid orally administered dosage forms [26]. The interpretation of the results from the texture profile of formulation was performed according Kulawik-Pióro et al. [27].

The cohesiveness refers to a formulation’s ability to maintain its original texture after being subjected to mechanical stress. A value close to 1 indicates that the texture remains largely unchanged or is quickly restored after mechanical action. As shown in Table 2, formulations B3, D3, E3, and F3 exhibit a cohesiveness close to 1. Additionally, formulations containing HPMC (A), MC (B), and HPC (F) demonstrated high compressibility compared to others. The most adhesive formulations were F3, A2, and D1. Considering the future preparation of the formulations involving solubilization in PG, we compared the mutual adhesiveness of samples from series 3. It was found that the adhesiveness increased in the following order: B3 < C3 < D3 < A3 < E3 < F3.

### 2.4. Mucoadhesion Test

The mucoadhesive capacity of oromucosal preparations is crucial for ensuring the effectiveness of the contained active pharmaceutical ingredients. If a dosage form is not formulated to allow the drug to remain on the oral mucosa for a sufficient time, saliva will wash it away, limiting its oromucosal effect.

The current literature categorizes methods for evaluating mucoadhesion into direct and indirect approaches [28]. Direct methods involve measuring the force or time required to detach a formulation from a mucosal membrane or a mucosa-mimicking substrate. On the other hand, indirect methods assess properties such as viscosity, which influence the adhesion process with respect to the mucosal membrane.

The mucoadhesive ability of the prepared pastes was tested on a gelatin substrate. The first samples were applied to a 6.6% gelatin film, for the preparation of which only purified water was used. However, these substrates completely disintegrated after application of the samples and wetting with the artificial saliva and tempering to a body temperature. Therefore, a higher concentration of gelatin (12.5%; *w*/*w*) and the addition of glycerol were chosen for the preparation of the substrate, to prevent its disruption by heat. After the samples were applied to the gelatin substrate cutouts, they were remoistened with the artificial saliva (0.5 mL) and allowed to temper for 1 h at 36.5 ± 0.5 °C. Subsequently, the force required to detach the probe from the sample applied to the substrate was measured using a texture analyzer. The values of the mucoadhesion force are recorded in Table 3. The values range from 0.143 to 0.221 N, with an average value of 0.161 ± 0.017 N and a variability of 0.0003 N. The HPC-based formulation containing EO and DEX solubilized in PG showed the highest mucoadhesion ability. The other formulations showed very similar mucoadhesion ability. Interestingly, these values did not correlate with the adhesiveness values. It is true that the principle of testing differs (in the case of adhesiveness testing, the measuring probe penetrates the sample, and the adhesiveness was evaluated based on the textural profile); nevertheless, we expected some dependence.

The mucoadhesion test conducted using glycerogelatin films as a model substrate is innovative. While many studies utilize porcine, bovine buccal mucosa or similar biological substrates [29], glycerogelatin film allows for controlled conditions and reproducibility. At the same time, it is easy to prepare at the time of need, it does not need to be specially treated and cleaned, there is no risk of modification due to storage conditions, e.g., freezing, as in the case of biological material, and, at the same time, species variability is limited; finally, it is more economically accessible. This method aligns with findings from other research that advocates for standardized models to assess mucoadhesive properties effectively.

Most of the bioadhesive polymers used in dosage forms are either polyacrylic acid or cellulose derivatives [30]. The mucoadhesive properties of cellulose derivatives stem from their ability to form hydrogen bonds with mucins present in the mucosal surface [31]. This interaction is crucial for prolonging the residence time of the dosage form in the oral cavity.

Studies have shown that formulations such as three-layer HPC adhesion films containing dibucaine effectively treat oral ulcers, demonstrating significant adhesion to buccal mucosa for extended periods (60–120 min). Additionally, mucoadhesive gels based on CMC, HPC, and HPMC have been utilized as carriers for chlorhexidine, showing prolonged drug release suitable for treating gingivitis [30]. Laffleur et al. [32] designed mucosal films using HPMC and EC for delivering allantoin to patients with dry mouth. Ammar et al. [33] created HPMC- and EC-based mucoadhesive films with fluticasone propionate aimed at treating oral lesions.

Some sources claim that cationic polymers have shown better mucoadhesive properties [34]. This would imply that the most-mucoadhesive samples should be those with HPMC, HPC, and MC, which are cationic (depending on specific modifications). CMC and its salt are anionic, while HEC is generally neutral, but can exhibit anionic behavior in particular environments.

Although the results of a given test cannot be used to determine the most mucoadhesive formulation due to slight differences in the values, the use of texture analysis for direct quantifying mucoadhesion force can be considered as a precise measurement technique evaluating mucoadhesive strength.

### 2.5. Swelling-Ratio Measurement

The swelling and formation of a mucoadhesive gel structure are important properties of oromucosal medicines intended for the application to pathologically altered oral mucosa. The dosage form has to resist the continuous secretion of saliva and subsequent washing of the dosage form. An undesirable effect of swelling is the dissolution of the polymer and its transformation into a liquid form, which is then washed away, together with the drug by saliva, terminating oromucosal absorption.

Swelling is the expansion of polymer particles, creating internal pressure that separates adjacent particles. In the initial phase, when a polymer-based dosage form encounters an aqueous environment, solvent molecules diffuse into the polymer matrix, hydrating the chains and enhancing their mobility. The rate of solvent penetration significantly influences swelling behavior and drug release. In the second phase, the swollen polymer transforms into a colloidal system, marking the dissolution process where the polymer network expands to disperse incorporated drug particles [35,36].

**Figure 1:**
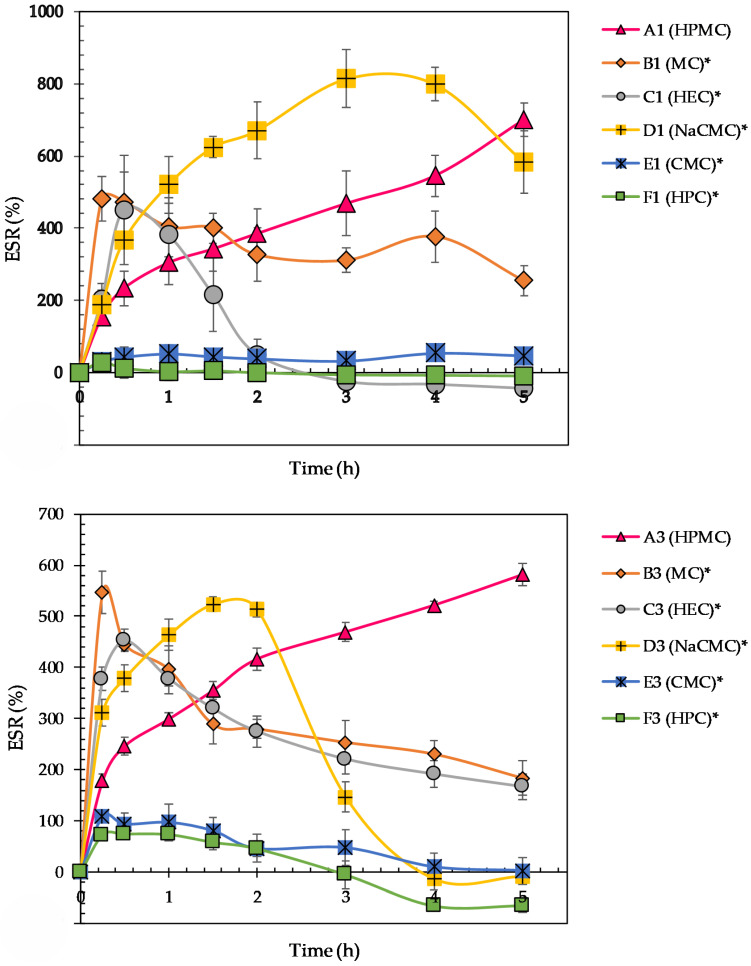
The comparison of the swelling ability of the formulations without essential oil (EO) (first series) and with EO and DEX solubilized in propylene glycol (PG) (third series). The difference in swelling ability of all formulations is statistically significant (*) compared to the reference samples A1 or A3 with HPMC.

**Figure 2:**
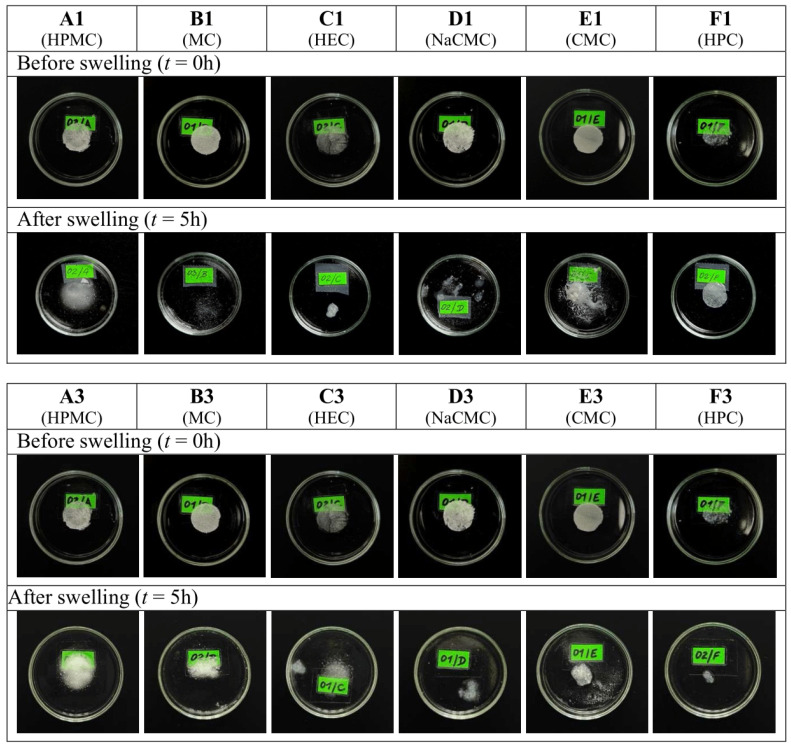
Macroscopic observation of changes in the sample formulations after 5 h of swelling in the artificial saliva.

The Equilibrium Swelling Ratio (ESR) was used to determine the degree of swelling and, indirectly, the mucoadhesion. The formulations were consistently flooded with a constant volume of artificial saliva, and after incubation at 37 °C, the weight gain or loss was determined at a specific time interval. After each measurement, the artificial saliva was replaced with a fresh volume to simulate fresh saliva secretion and movement in the oral cavity. The results are presented in Figure 1 and Figure 2. Since the formulations of the second series differed in the composition only in the content of two drops of peppermint oil compared to the formulations of the first series, only formulations A1 to F1 and A3 to F3 were subjected to the swelling ability test. Among the formulations studied, HPMC (ESR_max_ was 700 ± 46% after 5 h) emerged as the most optimal polymer for oromucosal administration, showing strong adhesion to the Petri dish and no significant reconstitution, even after five hours of swelling. PG significantly (*p* < 0.05) retarded the swelling ability of the above polymer (ESR_max_ was 581 ± 22% after 5 h) and showed strong adhesion to the Petri dish. Therefore, a presumption exists for the prolonged application of the HPMC formulation with PG additive. The formulations with NaCMC showed significant swelling ability (ESR_max_ was 814 ± 80% after 3 h) in a shorter time, compared to formulations with HPMC (ESR_max_ was 468 ± 90% after 3 h), but they started to reconstitute and wash away with additional artificial saliva after 3 h. They also did not show strong adhesion to the Petri dish, indicating a disadvantage for the oromucosal administration. The other formulations (with MC, HEC, CMC, or HPC) had worse swelling ability compared to the previous two polymers. They either exhibited extremely poor swelling ability, e.g., CMC (ESR_max_ was 54 ± 6% after 4 h), HPC (ESR_max_ was 28 ± 12% after 15 min), or they started to reconstitute and wash away too quickly with the addition of the artificial saliva, e.g., MC and HEC, which started to reconstitute after 1 h.

### 2.6. DEX–Excipient Compatibility

Fourier-transform infrared (FT-IR) spectroscopy is a useful analytical technique for evaluating the compatibility of active ingredients and excipients in pharmaceutical products, by analyzing chemical changes in functional groups.

**Figure 3:**
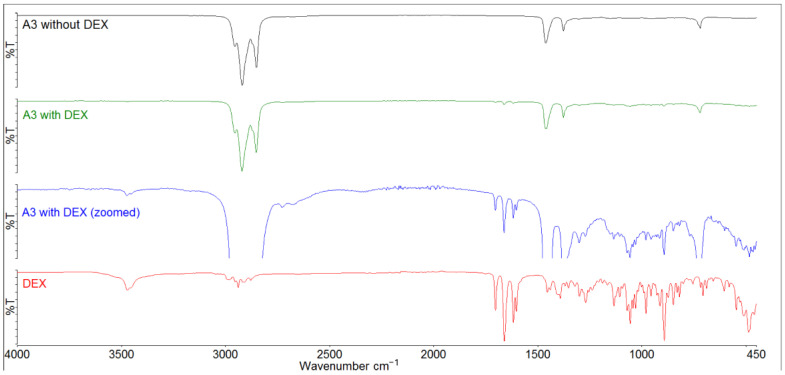
FT-IR spectra of DEX (red), formulation A3 with DEX (green), zoomed spectra of formulation A3 with DEX (blue), and formulation A3 without DEX (black).

Figure 3 showcases the FT-IR spectrum of pure DEX and formulation A3 with and without DEX (for FT-IR spectra of B3-F3, see Supplementary Information). The FT-IR spectrum of DEX exhibits a broad double peak around 3400–3500 cm^−1^, corresponding to the O–H stretching of hydroxyl groups. Peaks around 2850–3000 cm^−1^ correspond to various aliphatic C–H stretches. The prominent absorption bands around 1600–1700 cm^−1^ are attributed to the C=O stretching at C_20_ (1704 cm^−1^) and C_3_ (1661 cm^−1^), and C_1_=C_2_ and C_4_=C_5_ stretches at the A-ring of DEX (1617 and 1603 cm^−1^). Bands near 1000–1300 cm^−1^ are associated with various C–O stretching vibrations of hydroxyl groups. The strong absorption peak at 892 cm^−1^ corresponds to the vibration of 1,4-diene-3-ketone moiety. Similar FT-IR spectra for DEX were reported by Santos [37]. It was observed that DEX remained unchanged and stable in all formulations during storage, as almost all abovementioned characteristic absorption bands are present in the spectra of DEX containing formulations. The only exception is the absorption bands of C-H stretches, which overlap with strong absorption peaks originating from liquid paraffin.

FT-IR spectra indicate that DEX remains stable in the presence of excipients within dosage forms; however, its compatibility after oromucosal application in an aqueous environment remains uncertain. Although DEX is only slightly soluble in water [38], the presence of water increases the potential for incompatibilities. As a result, after the oromucosal application of DEX, we must consider not only the potential loss of the drug due to ingestion, but also the possibility of some degradation of the drug. Matter et al. [39] identified up to 13 degradation products of DEX in phosphate-buffered saline. It is important to note that their research involved testing DEX in implants designed for sustained drug release, which included in vitro release studies conducted over several days. Santos et al. [37] explored the compatibility of DEX with traditional excipients, primarily used as fillers in oral solid-drug formulations, using FT-IR, X-ray diffraction, and differential thermal analysis (DTA). Their results suggest potential interactions between DEX and the excipients, particularly due to heat, as these interactions were only observed using DTA. Based on FT-IR spectroscopy, Long et al. [40] admit the existence of DEX in polyvinyl alcohol hydrogel matrix with possible interactions between drug, crosslinker and polymer. To enhance the stability of DEX, one potential approach is the development of DEX conjugates [11].

### 2.7. In Vitro Drug-Release Study

The “availability” of DEX from the formulations was studied using an in vitro release test. The amount of released DEX was measured at specific time intervals (0.25 h, 0.5 h, 1 h, 1.5 h, 2 h, 3 h, 4 h). The membrane was utilized for the drug release experiments, allowing only the release through passive diffusion, and simplifying mathematical operations in predicting pharmacokinetics. To ensure realistic conditions, the system was maintained under “sink” conditions. This prevents passive diffusion from being affected by transfer in the opposite direction, provided that the amount of permeate does not exceed 10% of its degree of saturation in the acceptor medium (PBS 7.4) [41]. To predict the pharmacokinetics during a drug release, several mathematical models were developed. The key is to determine the permeation coefficient (K_p_) for characterizing drug release from dermal dosage forms. Additionally, the flux (Jss), representing the amount of substance passing through a unit area into the acceptor medium per unit time (μg.cm^−2^.h^−1^), was studied. Flux Jss (μg·cm^−2^ h^−1^) was determined by calculating the slope of the linear portion of the cumulative amount (µg·cm^−2^) over time. The permeation coefficient (K_p_) was calculated as a ratio of flux (Jss; μg.cm^−2^.h^−1^) and initial drug concentration (Ci; µg) [42]. According to Fick’s first law of diffusion, the flux is directly proportional to the concentration gradient and the permeation coefficient. The basic drug-release parameters, together with coefficient of determination (R^2^) for the kinetic models, are recorded in Table 3.

**Figure 4:**
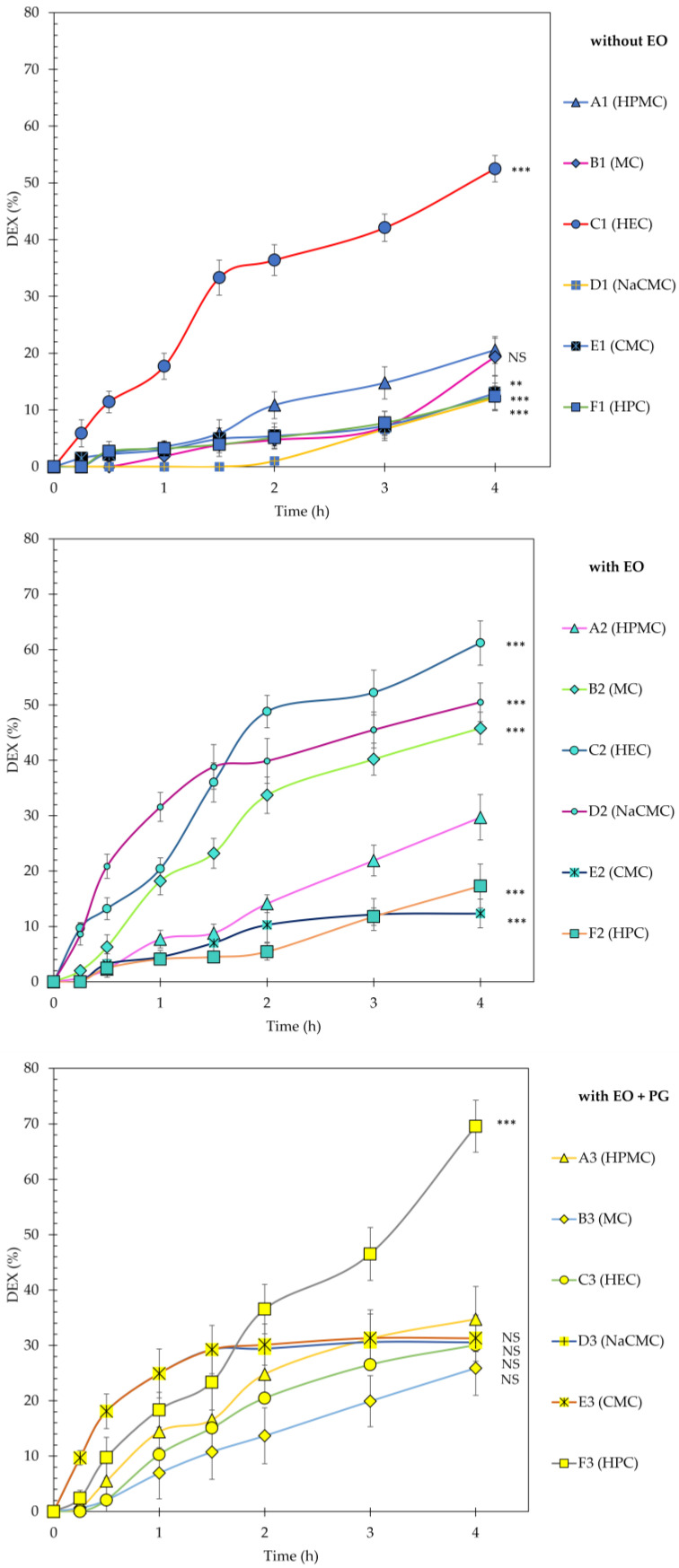
In vitro release profiles of DEX from the formulations without essential oil (EO), with essential oil (EO), and with essential oil (EO) and DEX solubilized in propylene glycol (PG). A1, A2 and A3 were used as the reference samples in the series of formulations being compared. NS indicates a non-significant difference, two asterisks (**) a significant difference at a high level with *p* ≤ 0.01, and three asterisks (***) a significant difference at a very high level with *p* ≤ 0.001.

In any case, the basis for determining drug release kinetics is the liberation curve, i.e., tracking drug release (%) as a function of time t (Figure 4). Mathematical interpretation of five pharmacokinetic models included zero-order, first-order, Higuchi, Korsmeyer–Peppas, and Hixson–Cowell. DEX was predominantly released from the formulations by the Korsmeyer–Peppas kinetics model (C1, E1, F1, C2, E2, E3) or by zeroth-order kinetics (A1, D1, A2, F2, A3, F3). To a lesser extent, DEX was released according to Higuchi’s model (B2, D2, C3, D3), and only sporadically by first-order kinetics (B1 and B3). The mechanisms of DEX release from the formulations involve complex interactions between diffusion and erosion processes. The Korsmeyer–Peppas model indicates varied transport mechanisms. DEX likely diffuses through a hydrated gel layer formed around the polymer matrix or is released as the polymer matrix erodes, while zeroth-order kinetics suggests controlled and sustained release profiles beneficial for therapeutic applications [43].

**Figure 5:**
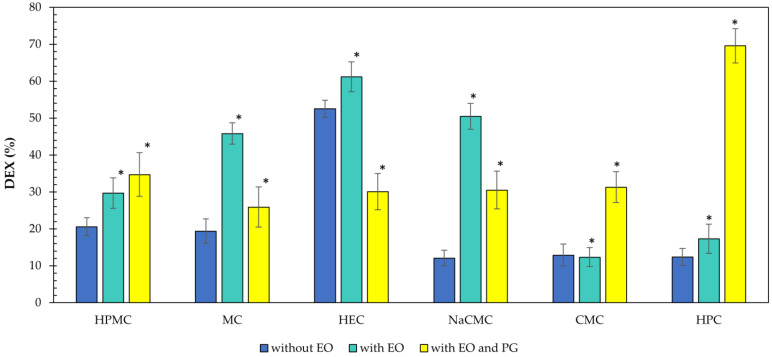
The amount of DEX (%) released after 4 h during in vitro release study from the formulations. The asterisk (*) indicates a significant difference with respect to the corresponding reference sample from series one, without EO and PG. The formulations without EO (blue), with EO (green), with EO and PG (yellow).

The addition of peppermint essential oil as a penetration promoter was confirmed to be statistically extremely significant (*p* < 0.0001) in most cases (HPMC, MC, HEC, NaCMC, and HPC). However, peppermint essential oil had a negligible or even negative effect on DEX release from the formulation with CMC, with statistically insignificant difference (*p* > 0.05) compared to reference without EO. The solubilization of DEX in PG caused a statistically significant increase in the drug release from the formulations containing HPMC, MC, NaCMC, CMC, and HPC compared to references without EO (in Figure 5, the comparison of blue versus yellow bars). There was also a significant increase compared to the corresponding samples containing EO (in Figure 5, the comparison of green versus yellow bars), but only in samples with HPMC, CMC, and HPC. Thus, in the present formulations, the combination of the penetration enhancers EO and PG acts synergistically. However, this change appears to be composition-dependent, since in the formulations containing MC, HEC and NaCMC, the same pair of penetration enhancers causes the opposite effect, namely a decrease in the amount of DEX released after 4 h.

Some formulations provided rapid drug release, but their mucoadhesion or behavior in an artificial saliva, key criterions for successful oromucosal application, were judged to be ineffective; e.g., the formulations with HEC, HPC or NaCMC.

Our findings lead to the conclusion that the type of the polymer chosen in the formulation can significantly affect the mucoadhesive and swelling abilities of the formulation. Using texture analysis, the highest mucoadhesion and adhesiveness was evaluated for HPC-based formulation containing EO and PG. However, despite this result, this formulation cannot be considered as the most suitable, which can be concluded based on the swelling test. Figure 2 shows the significant weight loss or degradation of this sample in the artificial saliva environment over a period of 5 h. Significant swelling ability was observed for HPMC-based formulations. These samples did not degrade over the 5 h, and they were increasing in volume.

The supportive effect of essential oils on drug release is documented by several studies [44]. The beneficial effects of essential oils on drug penetration are well-established, particularly in the context of dermal and transdermal applications. Their positive impact on drug absorption through the buccal mucosa can be attributed to interactions with the intercellular domains of proteins. This interaction induces conformational changes in the proteins and enhances the partitioning of the drug [45]. As in dermal applications, they temporarily reorganize the stratum corneum; similarly, in buccal applications, their action may lie in reorganization of the squamous stratified epithelium. Peppermint essential oil, together with others such as clove oil, tea tree oil, thyme oil, cinnamon oil, citrus oil, bergamot oil, and lavender oil, belong to the most widely studied and most used in dentistry [46]. Currently, the primary categories of drug release/absorption enhancers used for the oromucosal administration include fatty acids, surfactants, cholates, lauric acid, and alcohols [47]. The results of our study confirm that we can safely include essential oils among them. In addition, peppermint essential oil also has its own therapeutic effects, especially antiviral and antibacterial. The antiviral activity mechanism seems to stem from the inhibition of viral replication. Due to their hydrophobic nature, essential oils likely target the lipids in the bacterial plasma membrane or mitochondria, functionally disrupting these structures by enhancing proton permeability, leading to their antibacterial activity [48]. This is also why peppermint EO is often added to cosmetic products for dental hygiene and to suppress halitosis [49,50].

Generally, PG is a humectant commonly used to improve texture and other physical properties of semisolid drug-dosage forms [51]. Moreover, PG acts as a solubilizer and a penetration enhancer, promoting the diffusion of mainly hydrophobic drugs through hydrophilic matrices [52]. Our primary aim was not to influence drug release by its addition, but to provide simplified weighing of small amounts of DEX by dissolving it in PG in the form of stock solution. Figure 5 shows that the presence of PG in the formulation had a beneficial effect on the release of DEX from formulations containing HPMC, CMC and HPC. In the case of the latter formulation, the increase in DEX release after 4 h was up to 5.6-fold, compared to the reference sample without EO and PG.

The findings of this study are comparable to those of [53], who worked on the development of generic corticoid semisolid formulations. They emphasized the importance of optimizing formulation components to ensure comparable bioavailability and efficacy with branded products. Like our results, their study highlights how the selection of penetration enhancers, like PG and essential oils, plays a crucial role in modulating the drug-release and liberation profiles of corticosteroid formulations. This supports the conclusion that both excipient choice and formulation strategy are key to achieving efficient drug delivery.

The HPMC-based formulation with peppermint essential oil exhibited the best balance between DEX release and mucoadhesion, making it a promising candidate for oromucosal drug delivery. This finding agrees with the study by Sakuramoto et al. [54], who demonstrated that combining strong mucoadhesion with efficient drug release is essential for oromucosal applications, particularly in treating conditions like stomatitis. The sustained release observed in our HPMC formulation, along with good mucoadhesive properties, makes it suitable for such applications. 

In conclusion, this in vitro release study demonstrated that DEX release from hydrophilic matrices was primarily diffusion controlled, as described by the Korsmeyer–Peppas model. Both peppermint essential oil and propylene glycol were found to be effective penetration enhancers, though their effects were polymer-dependent. These results underscore the importance of optimizing both the polymer matrix and absorption enhancers to achieve efficient drug delivery, in oromucosal applications.

The HPMC-based formulation achieved the best swelling, and the addition of mint essential oil and PG promoted the drug release 1.7-fold, which means that using 1% DEX (*w*/*w*) dissolved in PG to simplify the weighing of DEX was a good choice (*p* < 0.05).

### 2.8. Irritation Potential

The prepared formulations are supposed to be used in the treatment of diseases of the oral mucosa. The HET-CAM test was used to determine the safety of their application. The same testing protocols were also applied to various oral products, including dental adhesive agents [55], sublingual nanocapsules [56] and hydrogels intended for the treatment of oral carcinoma [57,58]. This test is recommended by the Interagency Coordinating Committee on the Validation of Alternative Methods (ICCVAM) Recommended Test Methods (NIH Publication No. 10-7553—2010) to reduce suffering, pain, and distress in animals, to replace living organisms, and to minimize the number of animals used for testing. This test provides very relevant information, and therefore replaces the Draize test on rabbits, which involved direct application of substances to the eyes of these animals and was associated with significant pain and distress [59]. It also adheres to the 3Rs principle—Reduce, Refine, and Replace—as outlined by Russell and Burch (1959) [60].

**Figure 6:**
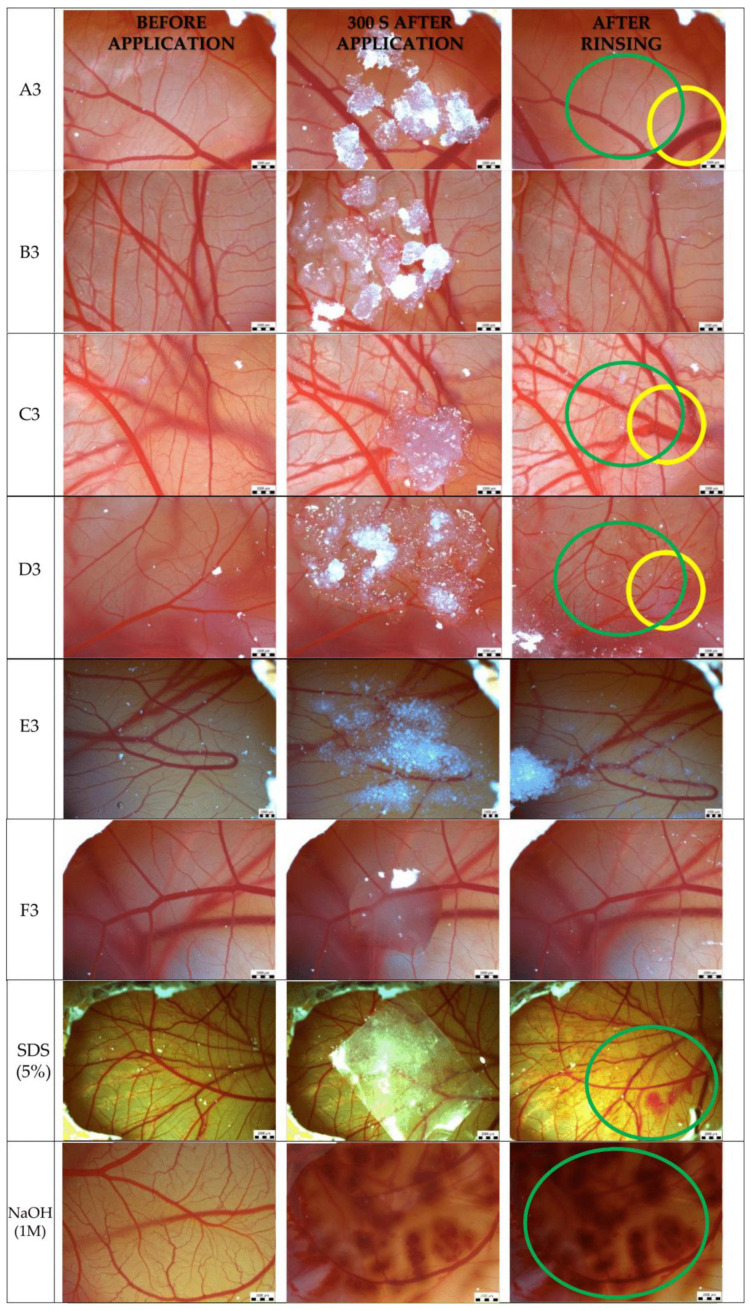
The microscopic observation of the chorioallantoic membrane after application of the examined formulations (green circles—hemorrhage, yellow circles—vasodilatation).

The formulations containing DEX, peppermint essential oil, and PG (third series, i.e., A3-F3) were tested. A total of 0.1 M aqueous solution of NaOH or 5% (*w*/*w*) SDS in soft white paraffin was used as positive control, showing all tested harmful effects—lysis of vessels, hemorrhage, and coagulation (Figure 6, Table 4). SDS in soft white paraffin was applied on CAM using parafilm to facilitate its removal. The appearance of the membrane after the application of the tested formulations at various times is depicted in Figure 6. The results show that the application of the tested formulations (A3, C3, and D3) leads to hemorrhage (marked in green circles). The same formulations (A3, C3 and D3) additionally caused vasodilatation (marked in yellow circles) at the site of the application and in its surroundings. According to irritation classification time-dependent score, the samples B3, E3, and F3 have a score of 0, which means they are non-irritant and samples A3, C3, D3 have a score of 3, which represents weak or slight irritation. We can conclude that A3, C3, and D3 can be classified as weak or slight irritants. On the other hand, it was found that three of the tested formulations with polymers MC, CMC and HPC (B3, E3, and F3) do not affect blood vessels.

Liquid paraffin and white soft paraffin were utilized as the constituents in all formulations. Additionally, DEX, peppermint essential oil, and PG were incorporated in each of the tested formulations. Therefore, we assume that the differences in irritation potential of the formulations are based on the content of a given polymer. The results indicate that white soft paraffin, liquid paraffin, DEX, or peppermint oil do not significantly affect the vascular integrity of the chicken embryo’s CAM, as hemorrhage and vasodilation were observed in some formulations but not in others. This implies that any observed irritation effects are likely attributable to the polymer, its possible interaction with DEX or peppermint essential oil, or the changes in osmolarity and pH.

Notably, the formulation containing the polymer hydroxyethyl cellulose (HEC) (C3) induced hemorrhage in the CAM, likely due to its acidic nature. This formulation exhibited the lowest pH (3.98 ± 0.05) compared to the other formulations tested. Other formulations with pH 6.09 ± 0.05 (B3), 6.36 ± 0.05 (E3) and 5.86 ± 0.05 (F3) do not have an adverse effect. These findings correlate with those reported by Anbarasan et al. (2019) [62], who demonstrated that in situ hydrogels containing Carbopol 934P with pH values ranging from 6.18 to 6.3 did not exhibit any adverse effects.

Our results indicate that the formulation containing CMC (E3) does not affect vascularity compared to its sodium derivative NaCMC (D3). It is hypothesized that the presence of sodium ions alters the osmolarity of the formulations, which may lead to slight hemorrhage and vasodilation [63].

A very slight irritation effect, manifested as hemorrhage, was observed in formulations containing HPMC (A3). This finding contrasts with the results reported by Elkasabgy et al. [64] and Ortega [57], who demonstrated that solid powder formulations of HPMC 15 cp (Elkasabgy, 2014) [64] and HPMC (Ortega, 2023) [57] incorporated into hydrogels did not elicit any irritant effects on the CAM. The formulations utilized in those studies included additional substances that likely minimized the potential for irritation. Conversely, the combination of polymers, DEX, and essential oil, in the current study, may have contributed to an increased irritation response, which was evidenced by very slight bleeding observed in the CAM. Local toxicity of the oral mucosa can be attributed to several factors, including chemical irritation, non-physiological pH, osmolarity, and salt concentration [63].

The formulations exhibited either non-irritant or minimal effects on the chicken CAM, suggesting their compatibility with this sensitive biological model. The oral mucosa consists of multiple layers that serve protective functions. It can tolerate certain irritants more effectively than the CAM because of its thicker epithelial layer and the presence of underlying connective tissue [65]. It is anticipated that formulations demonstrating only slight irritant effects on the CAM will not adversely affect the oral mucosa. This implies that the tested in situ hydrogels will possess tissue compatibility without causing local irritation. Following application, patients are expected to experience no pain, inflammatory reactions, or tissue damage when using the hydrogel containing DEX and peppermint essential oil.

The performance of a polymer in artificial saliva is a direct reflection of its intrinsic physical and chemical properties, which are fundamental to its suitability for oromucosal applications. Mucoadhesion, for instance, relies heavily on the polymer’s ability to form strong hydrogen bonds or electrostatic interactions with the mucosal surface. While HEC and NaCMC are hydrophilic, their structural characteristics limit their capacity to maintain prolonged adhesion, resulting in insufficient retention on the mucosal surface.

Swelling behavior, a critical factor in oromucosal drug delivery, further highlights the differences between these polymers. Although NaCMC initially demonstrated notable swelling, its polymer matrix lacked the robustness to sustain its structure in artificial saliva, leading to rapid disintegration and washout. HPC exhibited even poorer swelling, disintegrating prematurely, which significantly undermined both its mucoadhesion and drug-release capabilities.

In stark contrast, HPMC excelled, due to its ability to swell in a controlled manner, forming a cohesive gel layer that maintained its integrity in the presence of saliva. This property not only enhanced its mucoadhesion, but also enabled a sustained and controlled release of the drug. These findings clearly underscore the decisive role of the polymer matrix in determining the success of oromucosal formulations. By highlighting the superior performance of HPMC compared to HEC, HPC, and NaCMC, our results strongly support the conclusion that the choice of polymer is pivotal in achieving the desired balance between mucoadhesion and sustained drug release.

The selection of HPMC as the most promising polymer for the formulation is supported by various test results. Firstly, the swelling test showed that the sample containing HPMC did not disintegrate even after 5 h in a saliva environment; instead, it continued to swell, and its ESR increased, as you can see in Figure 1. The HPMC-based formulation’s ESR_max_ was 700 ± 46% after 5 h. Therefore, it was evaluated as the most optimal polymer for buccal administration, showing strong adhesion to the Petri dish and no significant reconstitution, even after five hours of swelling. It is the only polymer that continued to swell after 4 h. Thus, it is provided as a solution for long-term drug delivery in the oral cavity. In terms of drug release, many formulations have been more successful than HPMC. But we decided to choose the criterion of swelling ability as the key criterion, as it is crucial for the formulation to remain in the oral cavity for as long as possible, and thus to be long-acting. In terms of in vitro DEX release, from the five formulations that did not contain HPMC, a higher amount of the drug was released within 4 h, but the swelling behavior, and visual inspection after 5 h in artificial saliva (Figure 2) indicate that those samples likely experienced washout or disintegration. Regarding the evaluation of adhesiveness through classical texture analysis, the samples from the third series containing HPMC and CMC were found to be the second most adhesive. The formulation based on HPC exhibited the highest level of adhesiveness, as confirmed by the mucoadhesion test on a gelatine substrate. However, the HPC-based formulation with EO demonstrated superior performance by the in vitro permeation test, due to the results of the swelling test, and HPC was ultimately deemed unsuitable as a polymer for the formulation. Our choice is supported by the study conducted by Bakhrushin et al., which tested the mucoadhesive properties of selected polymers, including those we used, such as HPC, HEC, and NaCMC, using two different procedures. Based on the results from both tests, HPMC and xanthan gum demonstrated the best mucoadhesive abilities.

Despite our best efforts to outline the various procedures and measurement methods employed in this research, certain limitations remain that could be addressed in future studies. The following aspects of our work present opportunities for improvement:

Swelling Behavior: to mitigate the issue of weight loss in samples caused by their removal, together with the dissolution medium, enhancements in the methodology for assessing swelling behavior are necessary.

Mucoadhesion Testing: in our study, we utilized a gelatine substrate to model buccal mucosa. However, this could be improved by employing more representative materials, such as mucin gel, or relevant animal models. This change would provide a more realistic assessment of mucoadhesion properties.

Permeation Studies: the use of PBS as a dissolution medium in permeation studies could be refined by substituting it with artificial saliva. This modification would better simulate the physiological conditions of the oral cavity and enhance the relevance of the findings.

FT-IR analysis of DEX stability: future studies should also monitor DEX stability within the formulation under conditions that mimic buccal application, specifically in a wet-saliva environment.

## 3. Conclusions

The study demonstrates that incorporating peppermint essential oil (EO) into formulations enhances in vitro drug release. Key findings indicate that the formulations exhibit acceptable pH levels and low irritancy risks, confirming their compatibility with DEX. The formulation with HPMC achieved the highest swelling in the artificial saliva, xpressed as ESR, which is a prerequisite for the gradual release of the drug over 4 h. Although HPC showed the highest mucoadhesion, the system breaks down on prolonged contact with an aqueous environment. The other polymers provided approximately the same values of mucoadhesion. The study underscores the potential of mucoadhesive systems to improve therapeutic efficacy by prolonging drug retention at the site of action, thereby addressing the limitations of conventional treatments.

## 4. Materials and Methods

Dexamethasone (dexamethasonum Ph. Eur. 11; CAS: 50-02-2; DEX), hydroxypropylmethylcellulose (hypromellosum; 1.2 kDa, CAS: 9004-65-3; HPMC), peppermint essential oil (menthae piperitae etheroleum; CAS: 8006-90-4), propylene glycol (propylene glycolum; CAS: 57-55-6; PG), and urea (CAS: 57-13-6) were purchased from Fagron (Olomous, Czech Republic). Liquid paraffin (paraffinum liquidum; CAS: 8042-47-5), potassium chloride (KCl, p.a.; CAS: 7447-40-7), potassium dihydrogen phosphate (KH_2_PO_4_, p.a.; CAS: 7778-77-0), sodium dihydrogen phosphate dihydrate (NaH_2_PO_4_·2H_2_O, p.a.; CAS: 13472-35-0), calcium chloride (CaCl_2_, p.a.; CAS: 10043-52-4), sodium sulfide (Na_2_S, p.a.; CAS: 1313-82-2), hydrochloric acid (HCl, p.a.; CAS: 7647-01-0), sodium hydroxide (NaOH, p.a.; CAS: 1310-73-2), and ethanol 96% (C_2_H_5_OH; CAS: 64-17-5) were purchased from Centralchem (Bratislava, Slovakia). White soft paraffin (vaselinum album Ph. Eur. 11; CAS: 8009-03-8) was sourced from Galvex (Banská Bystrica, Slovakia). Sodium chloride (NaCl, p.a.; CAS: 7647-14-5) and disodium hydrogen phosphate dodecahydrate (Na_2_HPO_4_·12H_2_O, p.a.; CAS: 10039-32-4) were obtained from Merck (Darmstadt, Germany). Ethanol 70% (C_2_H_5_OH; CAS: 64-17-5), and glycerol 85% (C_3_H_8_O_3_; CAS: 56-81-5) were procured from Mikrochem (Pezinok, Slovakia). Carboxymethyl cellulose (90 kDa, CAS: 9000-11-0; CMC), sodium carboxymethyl cellulose (250 kDa, CAS: 9004-32-4; NaCMC), and hydroxypropyl cellulose (850 kDa, CAS: 9004-64-2; HPC) were purchased from Dr. Kulich Pharma (Hradec Králové, Czech Republic). Hydroxyethyl cellulose (154 kDa, CAS: 9004-62-0; HEC) was from Hercules Aqualon (Wilmington, NC, USA). Methylcellulose (88 kDa, CAS: 9004-67-5; MC) was from Molar Chemicals (Halásztelek, Hungary). Ultra-purified water was prepared by Biosan Labaqua Bio (Vrhlika, Slovenia).

### 4.1. Preparation of DEX Formulations

DEX was dispersed in liquid paraffin by parts. Peppermint essential oil was added dropwise, and, after thorough mixing, the cellulose derivative was gradually incorporated. Finally, soft white paraffin was added, and the entire mixture was homogenized thoroughly.

In a third series of formulations (A3-F3), DEX was solubilized in propylene glycol (PG). The formulation process began with preparing a 1% (*w*/*w*) solution of DEX in PG by dissolving it over several hours with occasional shaking. Next, the DEX solution in PG was added to the polymer, and the mixture was homogenized thoroughly. Afterwards, peppermint essential oil was added dropwise, followed by liquid paraffin, and the entire mixture was homogenized thoroughly. The composition of all formulations is detailed in Table 5.

### 4.2. Measurement of the Actual Acidity

A total of 2.5 g of the sample was weighted into a 50 mL Erlenmeyer flask with a ground stopper. A total of 20 mL of boiling deionized water with a pH of 7.0 was added and shaken vigorously for 1 min. After cooling to 20 °C, pH was measured by pH meter with a needle electrode (pHenomenal, VWR International, Radnor, PA, USA).

### 4.3. Rheological Behavior

The rheology of the prepared formulations was determined using a rotational viscometer (Rheolab QC, Anton Paar, Graz, Austria). The measurements were taken 48 h after preparing the sample formulations. The sample was filled into the stationary cylinder up to the marked line, and then the rotating cylinder was inserted. The entire assembly was placed in the rheometer, maintaining a constant temperature of 25 ± 0.1 °C. During the measurement, tangential stress and dynamic viscosity readings were recorded every 10 s following a torque change, starting from the highest gear, and then proceeding to the lowest gear.

### 4.4. Texture Analysis

The textural properties of the prepared formulations were examined using a texture analyzer (TA.XT PLUS, Stable Micro Systems, Godalming, UK). A measuring probe with a 12 mm diameter was inserted at a speed of 1 mm/s to a distance of 5 mm into the 10 g sample, and the measurement was repeated in two cycles. The software (Texture Exponent 6.1.11.0, Stable Micro Systems, Godalming, UK) was used for data collection and analysis. Parameters such as hardness, compressibility, cohesiveness, and adhesion were determined from force-(g)-versus-time-(s) plots.

### 4.5. Mucoadhesion Test

Instead of buccal mucosa, a glycerogelatin film was used as a model mucosal substrate [29,66] to determine and compare the mucoadhesion force of the samples. A film was prepared from gelatin (12.5%; *w*/*w*) swollen in glycerol (53.12%; *w*/*w*) and purified water (34.37%; *w*/*w*). The film was allowed to cool in a thin layer on a Petri dish. Circles with a diameter of 13.5 mm were cut out of the gelatine, on which a thin layer of 0.100 g of the semisolid sample was then applied, with great precision. Before the test, the gelatin film with the sample was moistened with 0.5 mL of artificial saliva and allowed to temper in the incubator to a temperature of 36.5 ± 0.5 °C for one hour. The force (in grams) required to pull the measuring probe from the sample was determined using a texture analyzer. Before the actual pulling-away, the probe applied a force of 10 g to the sample for 10 s. The measured values were then converted to *F* (N) using the following relation (1):

(1) $$F=\frac{F_{1}\times g}{1000}$$

where *F*_1_ is the force measured in grams and *g* is gravitational acceleration (9.81 m.s^−2^).

### 4.6. Swelling-Ratio Measurement

The swelling ability, expressed as the equilibrium swelling ratio (ESR), was determined gravimetrically in artificial saliva.

Preparation of artificial saliva: the following compounds were dissolved in 900 mL of distilled water: sodium dihydrogen phosphate dihydrate (0.78 g), potassium chloride (0.40 g), sodium chloride (0.40 g), calcium chloride dihydrate (0.79 g), urea (1.00 g), and sodium sulfide (0.05 g). The solution was stirred until all compounds were dissolved. The distilled water was added to reach the total volume of 1000 mL. Finally, the solution was filtered.

Swelling test: approximately 0.1–0.2 g of the tested formulation was weighed simultaneously (*n* = 3) into preheated Petri dishes (37 °C ± 0.5 °C), to ensure that both the thickness and surface area of the samples were consistent for each measurement. A total of 5.0 mL of artificial saliva tempered to 37 °C ± 0.5 °C was then pipetted into each Petri dish. The Petri dishes were placed in a thermostat, set to the same temperature. At specified time intervals, the Petri dishes were taken out, the swelling medium was poured off, and the residue was removed with filter paper. After weighing the Petri dishes, the sample was again exposed to 5.0 mL of artificial saliva and placed in the thermostat. This process was repeated until the mass of the swollen sample became constant or began to decrease. The equilibrium swelling ratio (ESR) was calculated for each time, according to Equation (2):

(2) $$ESR\;\left(\%\right)=\frac{m_{t}-m_{t0}}{m_{t0}}\times 100\;\;$$

where *m_t_* is the weight of the swollen sample at time *t* and *m_t_*_0_ is the weight of the sample before swelling.

### 4.7. DEX–Excipient Compatibility

The compatibility of DEX with the other excipients in the formulations was analyzed using FT-IR spectroscopy in the mid-infrared range (4000–450 cm^−1^), using a Spectrum Two FT-IR spectrometer equipped with UATR accessory (Perkin Elmer, Waltham, MA, USA). Due to the detection limit of UATR, DEX formulations (A3-F3) were prepared using 10-times the original quantity of the drug. Formulations without DEX were used to record the background spectra. Prior to the analysis, powdered DEX and cellulose derivatives were dried in a desiccator. The UATR diamond crystal was cleaned between each recording using a cotton-bud moistened with isopropyl alcohol.

To record the FT-IR spectra, powdered samples were placed on the diamond crystal and then pressed with a pressure arm to ensure a good contact with the surface of the crystal. In case of liquid excipients, a drop of the excipient was dropped onto the crystal without applying any pressure to the sample. The samples of the formulations A3-F3 were smeared on the crystal and then gently pressed with the pressure arm, to create a uniform layer. The obtained spectra were then analyzed to identify characteristic absorption peaks of the functional groups of DEX. To evaluate the compatibility of DEX and other excipients, the recorded spectra of the formulations were compared with reference spectra of DEX and individual excipients.

### 4.8. In Vitro Release Study

Below are the specifications for the liberation apparatus and the procedure for the experiment: the apparatus consisted of eight static Franz diffusion cells made of glass, mounted on a magnetic plate. Each cell had a closed donor compartment where the sample membrane was stretched, and an acceptor compartment filled with the dissolution medium. The acceptor compartments were equipped with a shell tempered by a circulating thermostat, and their average volume was 28.0 ± 1.7 mL. They were loaded with magnetic stirrers that are synchronously rotated at a speed of 100 ± 5 rpm. The average diffusion area was 1.77 ± 0.1 cm^2^. The experiment was conducted under sink conditions. The acceptor compartment was filled with phosphate buffer solution (PBS) 7.4 and allowed to equilibrate at 37 ± 0.5 °C. The dialysis membrane (Spectra/Por^®^4, San Francisco, CA, USA) was stretched over the bottom of the donor compartments, with the sample accurately loaded. Then, 2.0 mL of artificial saliva was pipetted over the top of the sample. The donor compartments were then connected to the acceptor compartments, ensuring that the bottom of the membrane was in the contact with PBS. At prescribed time intervals, 1.0 mL of sample was withdrawn from each acceptor compartment, and the drug content of the sample was determined using UV spectrophotometry at λ_max_ = 238 nm against a blank taken from a Franz cell with a placebo sample. After each sample collection, 1.0 mL of PBS equilibrated at 37 ± 0.5 °C was added to the acceptor compartment. The experiment used cell^0^ as a blank (a sample of the formulation without the drug, applied to the membrane) and cells^1–7^ for parallel measurements of the formulation with the drug (*n* = 7).

A variety of kinetic models are employed to describe drug release profiles, each tailored to specific formulations and mechanisms. The zero-order kinetics model describes drug release at a constant rate, independent of concentration, ideal for controlled-release systems (M_t_ = M_0_ + k_0_t; M_t_ = amount of drug released at time t; M_0_ = initial amount of drug; k_0_ = release rate constant). First-order kinetics indicates that the release rate is proportional to the remaining drug concentration, commonly used in immediate-release formulations (lnM_t_ = lnM_0_ − k_1_t; M_t_ = amount of drug released at time t; M_0_ = initial amount of drug; k_1_ = first-order rate constant). The Higuchi model, based on Fickian diffusion, applies to homogeneous matrices (M = k_H_t^1/2^; M = amount of drug released; k_H_ = diffusion constant; t = time) and is suitable for matrix tablets and patches. The Korsmeyer–Peppas model accounts for both diffusion and erosion in complex release profiles (M_t_/M_max_ = k_K_t^n^; M_t_ = amount of drug released at time t; M_max_ = total amount of drug; k_K_ = kinetic constant; *n* = release exponent) and is often used for hydrogels. Lastly, the Hixson–Crowell model considers changes in surface area and shape during dissolution (M_t_^1/3^ − M_max_^1/3^ = k_H_t; M_t_ = amount of drug released at time t; M_max_ = total amount of drug released; k_H_ = rate constant; t = time), applicable for tablets undergoing surface erosion [67].

### 4.9. Irritation Potential

Fifty fertilized chicken eggs (Gallus gallus domesticus, Lohmann Brown breed) were acquired from a certified hatchery, Parovké Háje, in Slovakia. The surface of the eggs was gently washed and disinfected using 70% ethanol (Mikrochem, Pezinok, Slovakia). The eggs were then positioned horizontally in an incubator set at a temperature of 37.5 ± 0.5 °C with a relative humidity of 60–80% (River system, Campodarsego, Italy). After 24 h of incubation, 3 mL of albumin was extracted from the blunt end of each egg, using a needle and syringe. The resulting small hole was sealed with paraffin wax (Mikrochem, Pezinok, Slovakia). The eggs remained in the incubator under the same conditions until embryonic day 10. At that point, a small hole was cut into each eggshell, using scissors. Ten milligrams of each substance being tested was carefully placed on the surface of the CAM. For negative controls, 0.9% NaCl and white soft paraffin were used. The positive controls included 0.1 M NaOH in water and 1% SDS in soft white paraffin. The CAM was photographed with a digital camera (PROMICAM 3-3CP) using QuickPHOTO MICRO 3.2 software (Prague, Czech Republic) on an Olympus SZ61 stereomicroscope (Olympus, Tokyo, Japan) before application and at 30 s, 2 min, and 5 min after application. The tested substances were removed with a sterilized saline solution (0.9% NaCl, *w*/*w*), and the CAM was photographed again. Each substance was tested a minimum of six times. The results were evaluated using the S-Score model (severity score) by three independent scientists, to minimize subjective bias [61]. Approval for the animal protocol was not required, due to exemptions outlined in the legislation on the protection of animals used for scientific purposes (2010/63/EU).

### 4.10. Statistical Analysis

All experimental data are presented as the mean values ± standard deviation (S.D.). The results were analyzed using both one-way and two-way analysis of variance (ANOVA), followed by Tukey post hoc tests. The statistical analysis was performed using GraphPad Prism 10.0 software.

## Figures and Tables

**Table 1:** The physical characterization of the formulations.

Formulation	Difference in Composition	pH ± S.D.	Ƞ^+^_1_ ± S.D. (Pa.s)	Ƞ^−^_1_ ± S.D. (Pa.s)	ΔȠ_1_ ± S.D. (Pa.s)
A1	HPMC	6.14 ± 0.05	989.5 ± 19.8	1040.8 ± 20.8	51.3 ± 1.0
A2	HPMC + EO	6.09 ± 0.05	14.7 ± 0.3 *	19.8 ± 0.4 *	5.1 ± 0.1 *
A3	HPMC + EO + PG	6.07 ± 0.05	856.5 ± 17.1	1490.1 ± 29.8 *	633.6 ± 12.7 *
B1	MC	6.22 ± 0.05	1789.2 ± 35.8	1870.1 ± 37.4	80.9 ± 1.6
B2	MC + EO	6.13 ± 0.05	144.1 ± 2.9 *	9.7 ± 0.2 *	−134.4 ± 2.7 *
B3	MC + EO + PG	6.09 ± 0.05	828.6 ± 16.6 *	600.9 ± 12.0 *	−227.7 ± 4.6 *
C1	HEC	4.17 ± 0.05	1958.9 ± 39.2	2168.9 ± 43.4	210 ± 4.2
C2	HEC + EO	4.05 ± 0.05	51.4 ± 1.0 *	77.4 ± 1.5 *	26 ± 0.5 *
C3	HEC + EO + PG	3.98 ± 0.05	857.5 ± 17.2 *	604.3 ± 12.1 *	−253.2 ± 5.1 *
D1	NaCMC	6.45 ± 0.05	6.8 ± 0.1	4.3 ± 0.1	−2.5 ± 0.0
D2	NaCMC + EO	6.30 ± 0.05	24.5 ± 0.5 *	28.3 ± 0.6 *	3.8 ± 0.1
D3	NaCMC + EO + PG	6.31 ± 0.05	24.6 ± 0.5 *	63.1 ± 1.3 *	38.5 ± 0.8 *
E1	CMC	6.34 ± 0.05	21.4 ± 0.4	67.4 ± 1.3	46 ± 0.9
E2	CMC + EO	6.42 ± 0.05	7.3 ± 0.1 *	4.1 ± 0.1 *	−3.2 ± 0.1 *
E3	CMC + EO + PG	6.36 ± 0.05	23 ± 0.5	8.1 ± 0.2 *	−14.9 ± 0.3 *
F1	HPC	6.14 ± 0.05	13.8 ± 0.3	14.4 ± 0.3	0.6 ± 0.0
F2	HPC + EO	6.03 ± 0.05	2514.8 ± 50.3 *	80.9 ± 1.6 *	−2433.9 ± 48.7 *
F3	HPC + EO + PG	5.86 ± 0.05	1848.7 ± 37.0 *	803.9 ± 16.1 *	−1044.8 ± 20.9 *

Ƞ^+^_1_ viscosity measured at shear rate 6.45 s^−1^ at ascending torque; Ƞ^−^_1_ viscosity measured at shear rate 6.45 s^−1^ at descending torque; * *p* ≤ 0.05; the reference sample was A1 for A samples, B1 for B samples, etc.

**Table 2:** The main characteristics evaluated by texture analysis.

Formulation	Difference in Composition	Mucoadhesion ± S.D. (N)	Adhesiveness ± S.D. (g.s)	Compressibility ± S.D. (g.s)	Cohesiveness ± S.D.
A1	HPMC	0.159 ± 0.000	−24.65 ± 0.49	676.68 ± 13.53	0.87 ± 0.02
A2	HPMC + EO	0.145 ± 0.006 *	−39.98 ± 0.80 *	1722.58 ± 34.45 *	0.54 ± 0.01 *
A3	HPMC + EO + PG	0.154 ± 0.012	−27.39 ± 0.55 *	1634.30 ± 32.70 *	0.92 ± 0.02 *
B1	MC	0.143 ± 0.000	−8.55 ± 0.17	2251.33 ± 45.03	0.49 ± 0.01
B2	MC + EO	0.154 ± 0.000 *	−8.45 ± 0.17	1782.17 ± 35.64 *	0.71 ± 0.01 *
B3	MC + EO + PG	0.154 ± 0.005 *	−10.27 ± 0.21 *	1205.76 ± 24.12 *	0.98 ± 0.02 *
C1	HEC	0.158 ± 0.004	−36.16 ± 0.72	36.20 ± 0.72	0.94 ± 0.02
C2	HEC + EO	0.168 ± 0.004	−0.08 ± 0.00 *	30.90 ± 0.62 *	0.92 ± 0.02
C3	HEC + EO + PG	0.166 ± 0.002	−11.27 ± 0.23 *	249.75 ± 5.00 *	0.72 ± 0.01 *
D1	NaCMC	0.155 ± 0.001	−39.37 ± 0.79	410.86 ± 8.22	0.81 ± 0.02
D2	NaCMC + EO	0.158 ± 0.006	−0.5 ± 0.01 *	29.30 ± 0.59 *	0.94 ± 0.04 *
D3	NaCMC + EO + PG	0.151 ± 0.004	−14.89 ± 0.30 *	72.14 ± 1.44 *	1.04 ± 0.02 *
E1	CMC	0.157 ± 0.004	−0.05 ± 0.00	911.76 ± 18.24	0.84 ± 0.22
E2	CMC + EO	0.169 ± 0.016	−0.05 ± 0.01	110.51 ± 2.21 *	0.25 ± 0.01 *
E3	CMC + EO + PG	0.169 ± 0.000	−29.3 ± 0.59 *	173.81 ± 3.48 *	0.96 ± 0.02 *
F1	HPC	0.158 ± 0.003	−14.08 ± 0.28	1234.40 ± 24.69	0.44 ± 0.01
F2	HPC + EO	0.157 ± 0.005	−7.76 ± 0.16 *	2635.55 ± 52.72 *	0.47 ± 0.01
F3	HPC + EO + PG	0.221 ± 0.056	−76.79 ± 1.54 *	1121.86 ± 22.44 *	1.04 ± 0.02 *

Note: * *p* ≤ 0.05; the reference sample was A1 for A samples, B1 for B samples, etc.

**Table 3:** The coefficient of determination (R^2^) of various kinetic models, flux (Jss), and permeation coefficient (K_p_) determined by in vitro release study of DEX from the formulations.

	R^2^					Jss (μg.cm^2^.h^−1^)	K_p_ × 10^3^ (cm.h^−1^)
	0th Order	1st Order	Higuchi	Korsm.–Peppas	Hixs.– Crowell		
A1	0.9879	0.9873	0.9524	0.9810	0.9877	0.1887	1.22
B1	0.9902	0.9958	0.9868	0.9818	0.8536	0.1372	0.96
C1	0.9292	0.9609	0.9732	0.9745	0.9520	0.2724	2.47
D1	0.8457	0.8413	0.7061	0.4876	0.8428	0.0921	0.63
E1	0.9426	0.9353	0.8733	0.9624	0.9378	0.0836	0.51
F1	0.9536	0.9508	0.9053	0.9585	0.9518	0.1559	0.86
A2	0.9942	0.9910	0.9590	0.9860	0.9926	0.2307	1.49
B2	0.9337	0.9648	0.9856	0.9536	0.9556	0.2877	2.40
C2	0.9180	0.9538	0.9591	0.9665	0.9434	0.4511	3.14
D2	0.8169	0.8792	0.9263	0.9035	0.8594	0.2742	2.16
E2	0.8788	0.8856	0.9523	0.9699	0.8834	0.0914	0.73
F2	0.9571	0.9508	0.8893	0.9569	0.9530	0.0936	0.69
A3	0.9942	0.9910	0.9590	0.9860	0.9926	0.2307	1.49
B3	0.9930	0.9977	0.9886	0.9449	0.9965	0.1934	1.47
C3	0.9431	0.9622	0.9895	0.9112	0.9562	0.2516	1.72
D3	0.9168	0.9438	0.9729	0.9641	0.9356	0.1611	1.21
E3	0.6442	0.6670	0.7992	0.8690	0.6595	0.1085	0.80
F3	0.9876	0.9484	0.9593	0.9611	0.9686	0.4868	3.44

**Table 4:** Classification of the formulations based on the irritation potential according to Leupke’s scoring scheme [61].

Score Range	Irritation Category	Score Range	Samples
0–0.9	No irritation	0	B3, E3, F3
1–4.9	Weak or slight irritation	3	A3, C3, D3
5–8.9	Moderate irritation		
9–21	Strong or severe irritation	21	1 M NaOH, 5% SDS

**Table 5:** The composition of DEX formulations.

Formulation	A1	A2	A3	B1	B2	B3	C1	C2	C3	D1	D2	D3	E1	E2	E3	F1	F2	F3
DEX (g)	0.01	0.01	-	0.01	0.01	-	0.01	0.01	-	0.01	0.01	-	0.01	0.01	-	0.01	0.01	-
DEX dispersed in PG (1%) (g)	-	-	1.0	-	-	1.0	-	-	1.0	-	-	1.0	-	-	1.0	-	-	1.0
Mint essential oil (gtt.)	-	II	II	-	II	II	-	II	II	-	II	II	-	II	II	-	II	II
Hydroxypropyl Methyl Cellulose (g)	4.0	4.0	4.0	-	-	-	-	-	-	-	-	-	-	-	-	-	-	-
Methyl Cellulose (g)	-	-	-	4.0	4.0	4.0	-	-	-	-	-	-	-	-	-	-	-	-
Hydroxyethyl Cellulose (g)	-	-	-	-	-	-	4.0	4.0	4.0	-	-	-	-	-	-	-	-	-
Sodium Carbo- xymethyl Cellulose (g)	-	-	-	-	-	-	-	-	-	4.0	4.0	4.0	-	-	-	-	-	-
Carboxymethyl Cellulose (g)	-	-	-	-	-	-	-	-	-	-	-	-	4.0	4.0	4.0	-	-	-
Hydroxypropyl Cellulose (g)	-	-	-	-	-	-	-	-	-	-	-	-	-	-	-	4.0	4.0	4.0
White soft paraffin (g)	2.0	2.0	2.0	2.0	2.0	2.0	2.0	2.0	2.0	2.0	2.0	2.0	2.0	2.0	2.0	2.0	2.0	2.0
Liquid paraffin (g)	4.0	4.0	3.0	4.0	4.0	3.0	4.0	4.0	3.0	4.0	4.0	3.0	4.0	4.0	3.0	4.0	4.0	3.0

Note: “gtt” is an abbreviation derived from the Latin word “gutta”, which means “drop”.

## Data Availability

Data are contained within the article and Supplementary Materials.

## Supplementary Materials

The following supporting information can be downloaded at: https://www.mdpi.com/article/10.3390/gels11010026/s1, Figure S1: Rheological behavior of the formulations. Figure S2: Texture profiles of formulations A3-F3 and interpretation of the results from the texture profile of analysis test: adhesiveness = Area 3, cohesiveness = Area 2/Area 1, compressibility = Area 1. Figure S3: FT-IR spectra of the formulation B3 with DEX (red) and without DEX (black); Figure S4: FT-IR spectra of the formulation C3 with DEX (red) and without DEX (black); Figure S5: FT-IR spectra of the formulation D3 with DEX (red) and without DEX (black); Figure S6: FT-IR spectra of the formulation E3 with DEX (red) and without DEX (black); Figure S7: FT-IR spectra of the formulation F3 with DEX (red) and without DEX (black).
