# Supplementary figures and images for: In Situ Gelling Dexamethasone Oromucosal Formulation: Physical Characteristics Influencing Drug Delivery
Source: Gels. 2025 Jan 2;11(1):26. doi: 10.3390/gels11010026 (PMC11765448; doi:10.3390/gels11010026)

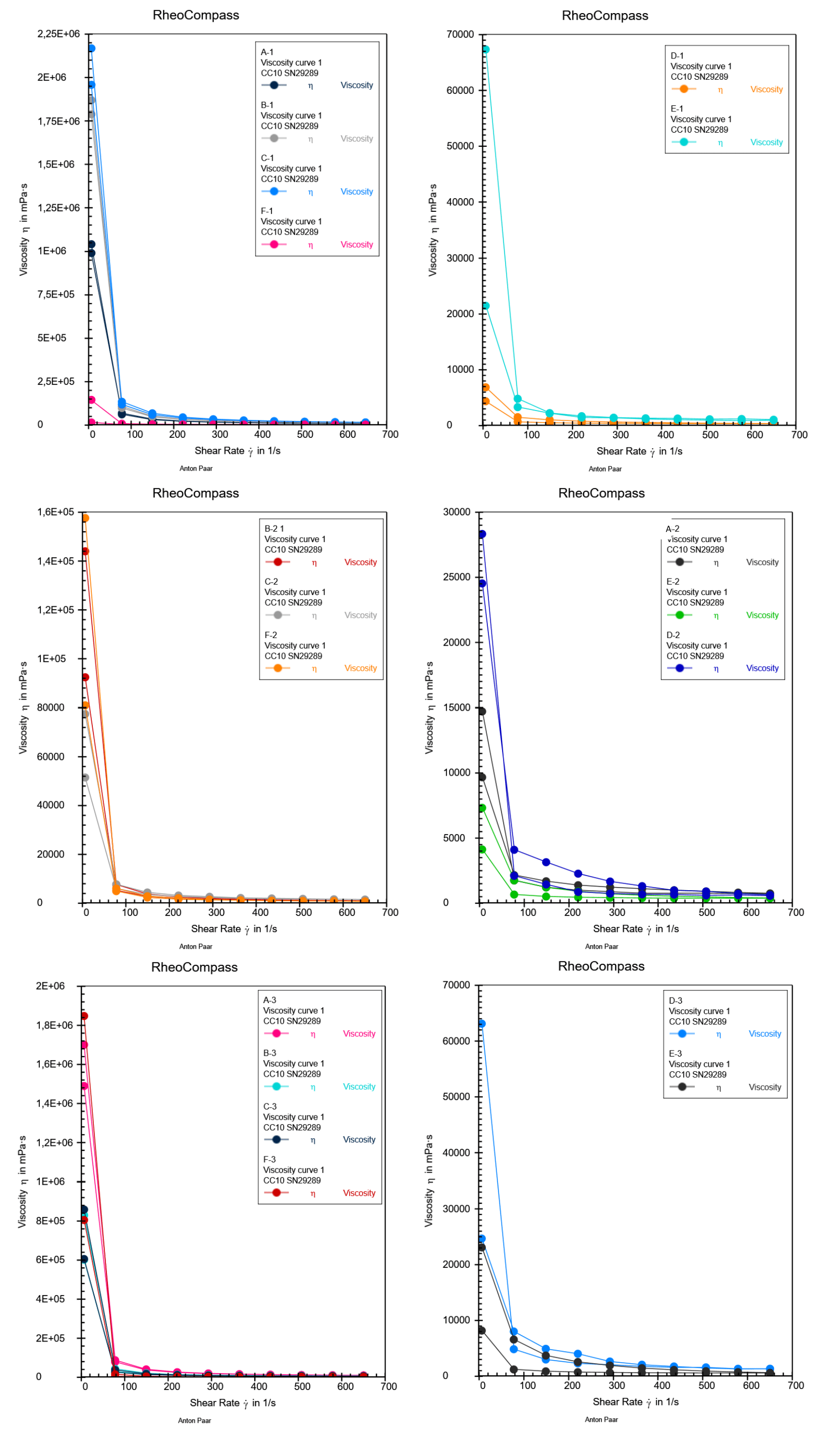

Supplement: Supplementary file 1 [file gels-11-00026-s001.zip › Figure S1.png]

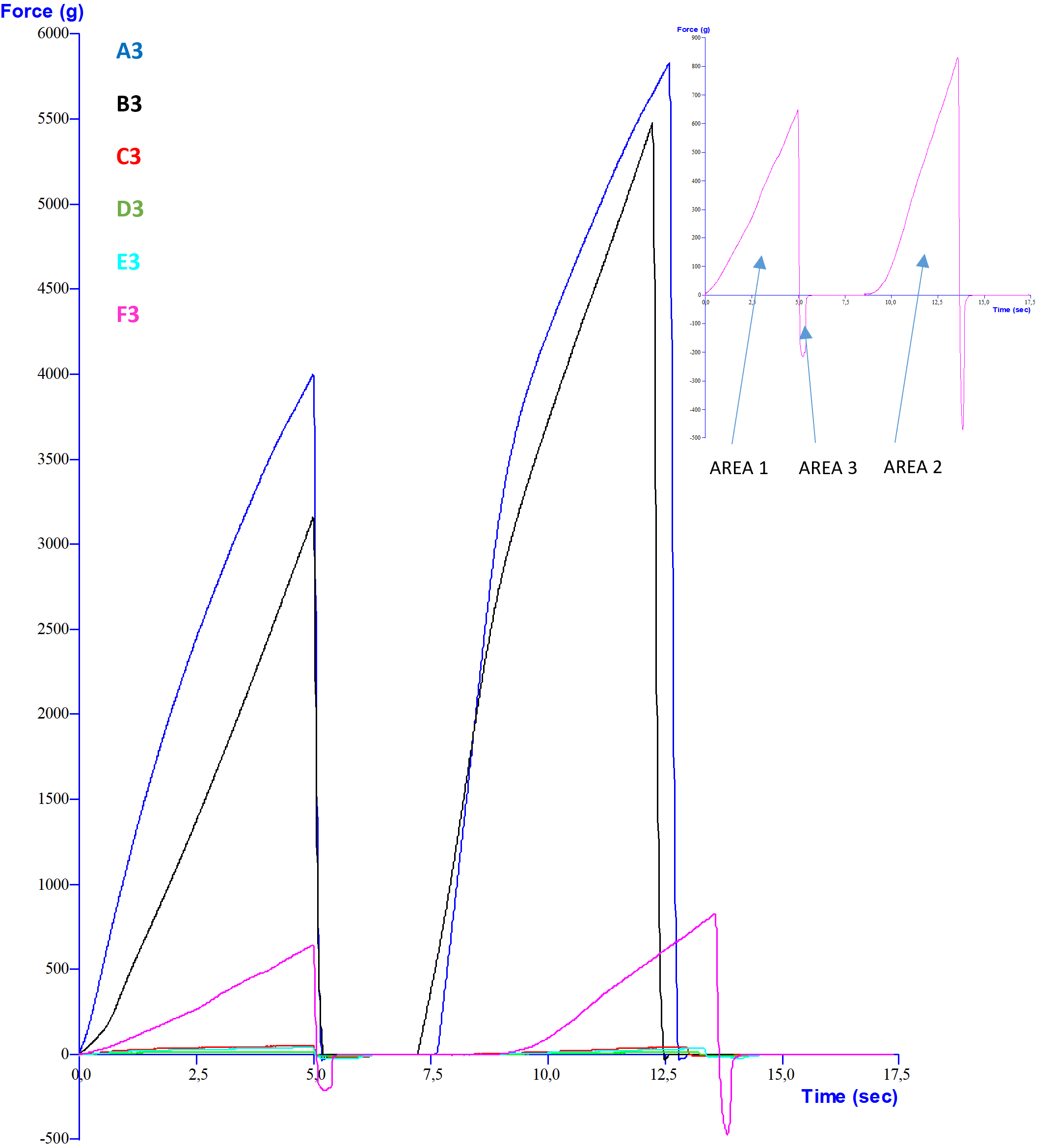

Supplement: Supplementary file 1 [file gels-11-00026-s001.zip › Figure S2.png]

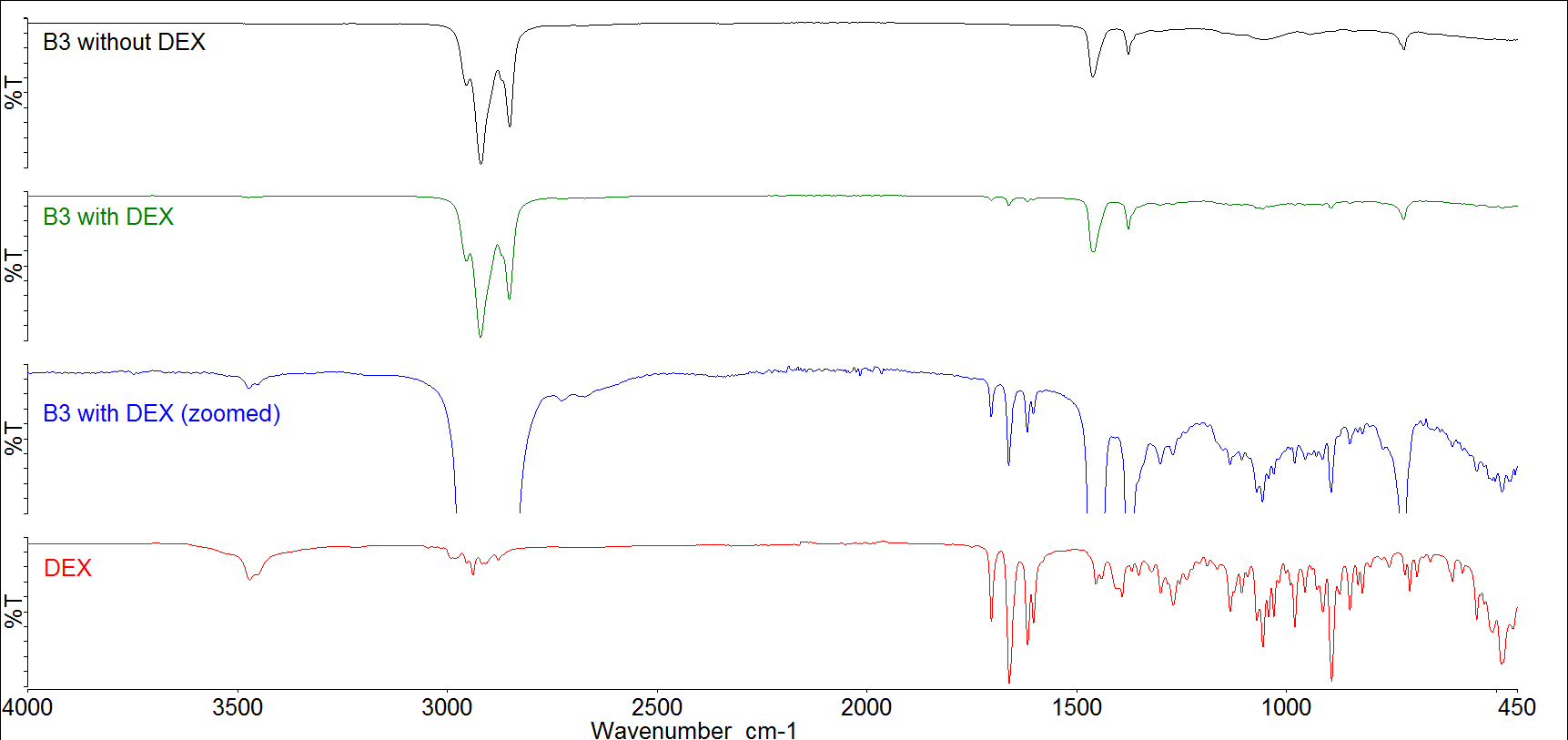

Supplement: Supplementary file 1 [file gels-11-00026-s001.zip › Figure S3.png]

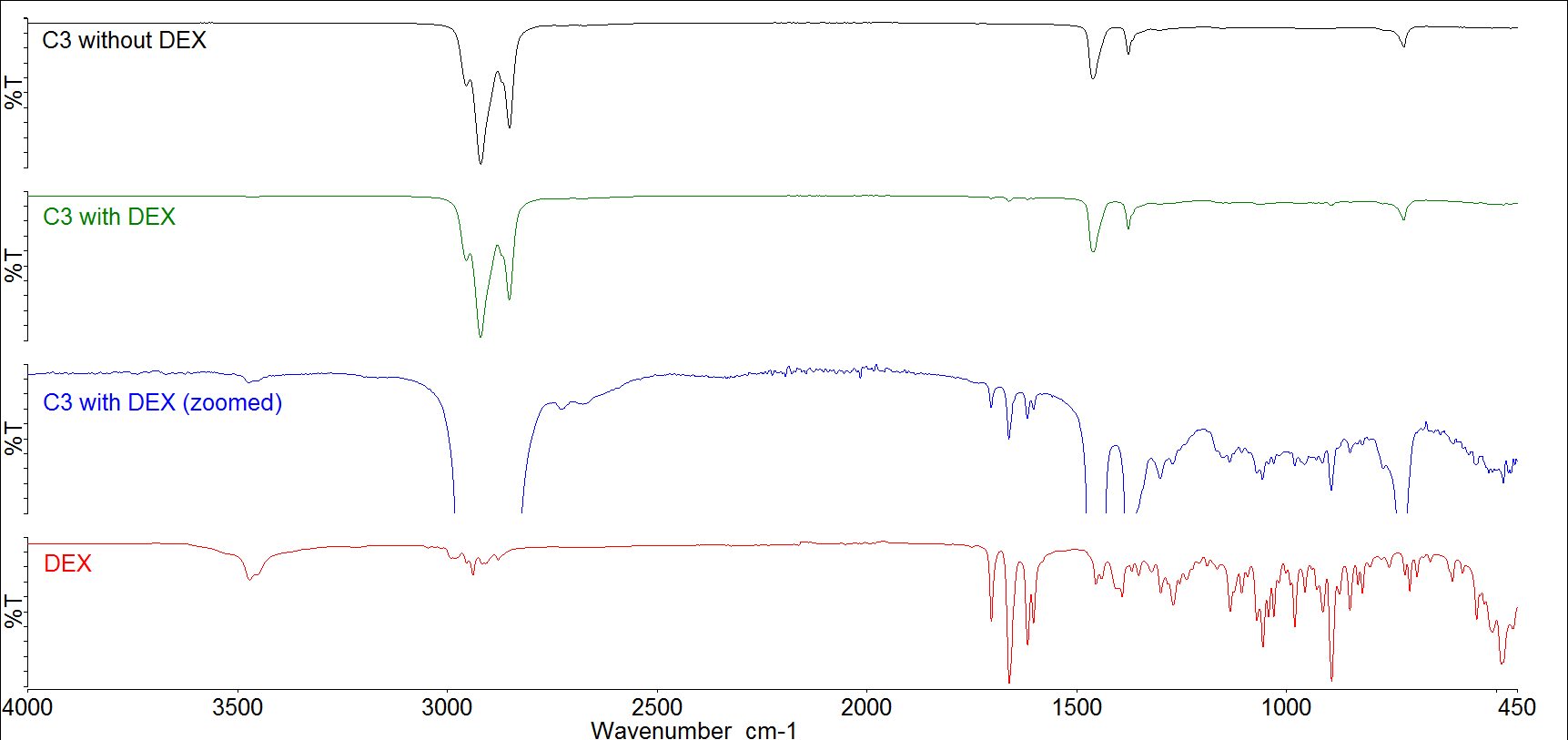

Supplement: Supplementary file 1 [file gels-11-00026-s001.zip › Figure S4.png]

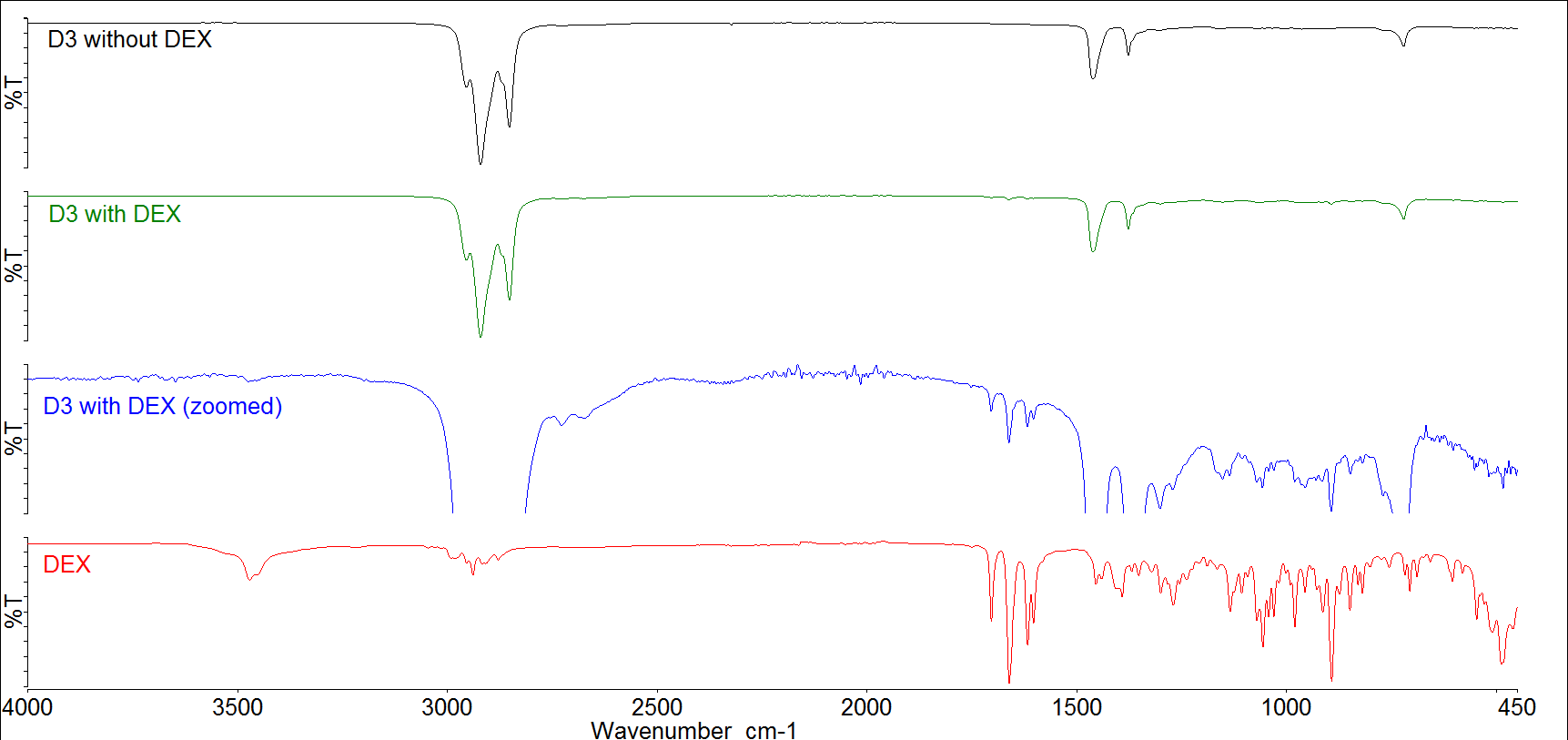

Supplement: Supplementary file 1 [file gels-11-00026-s001.zip › Figure S5.png]

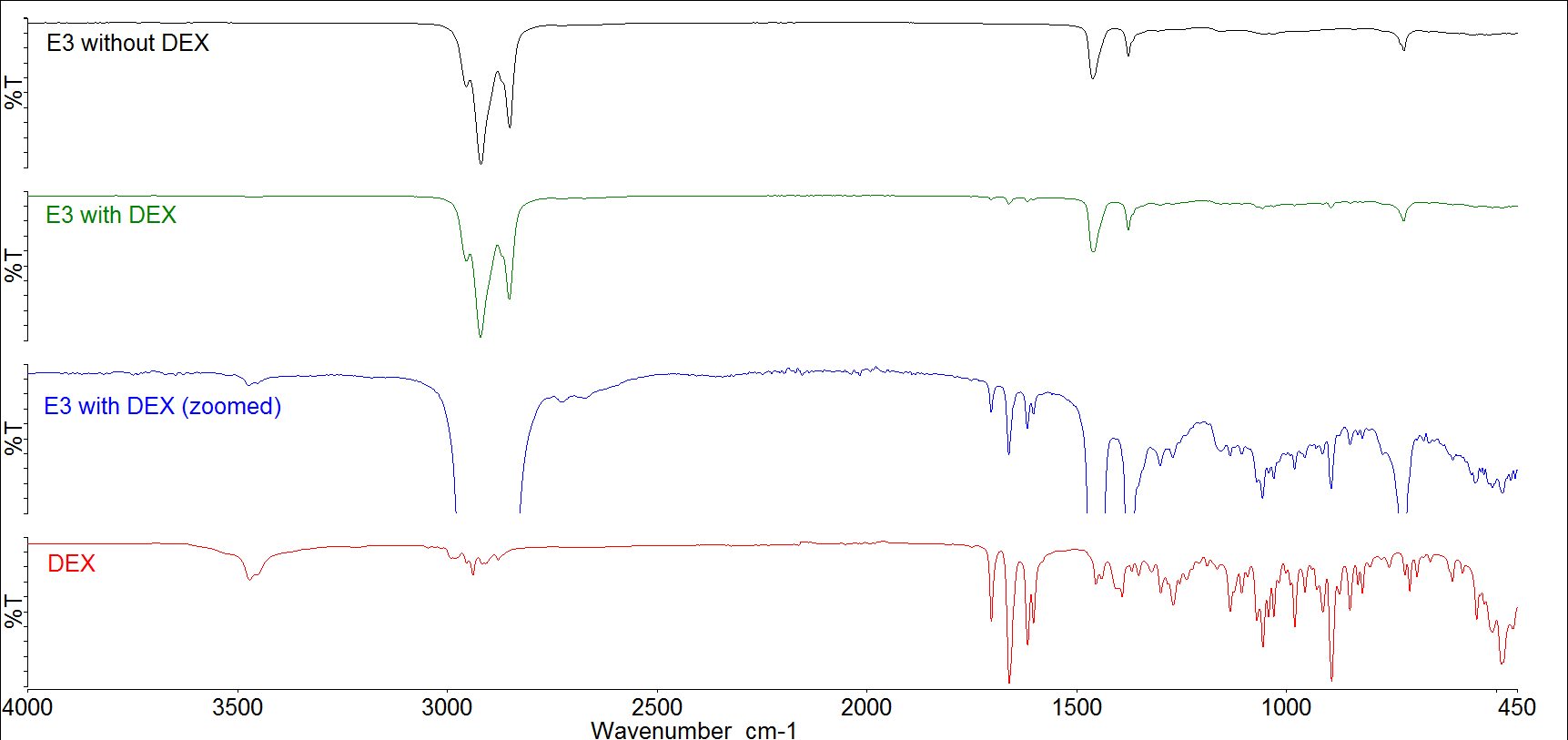

Supplement: Supplementary file 1 [file gels-11-00026-s001.zip › Figure S6.png]

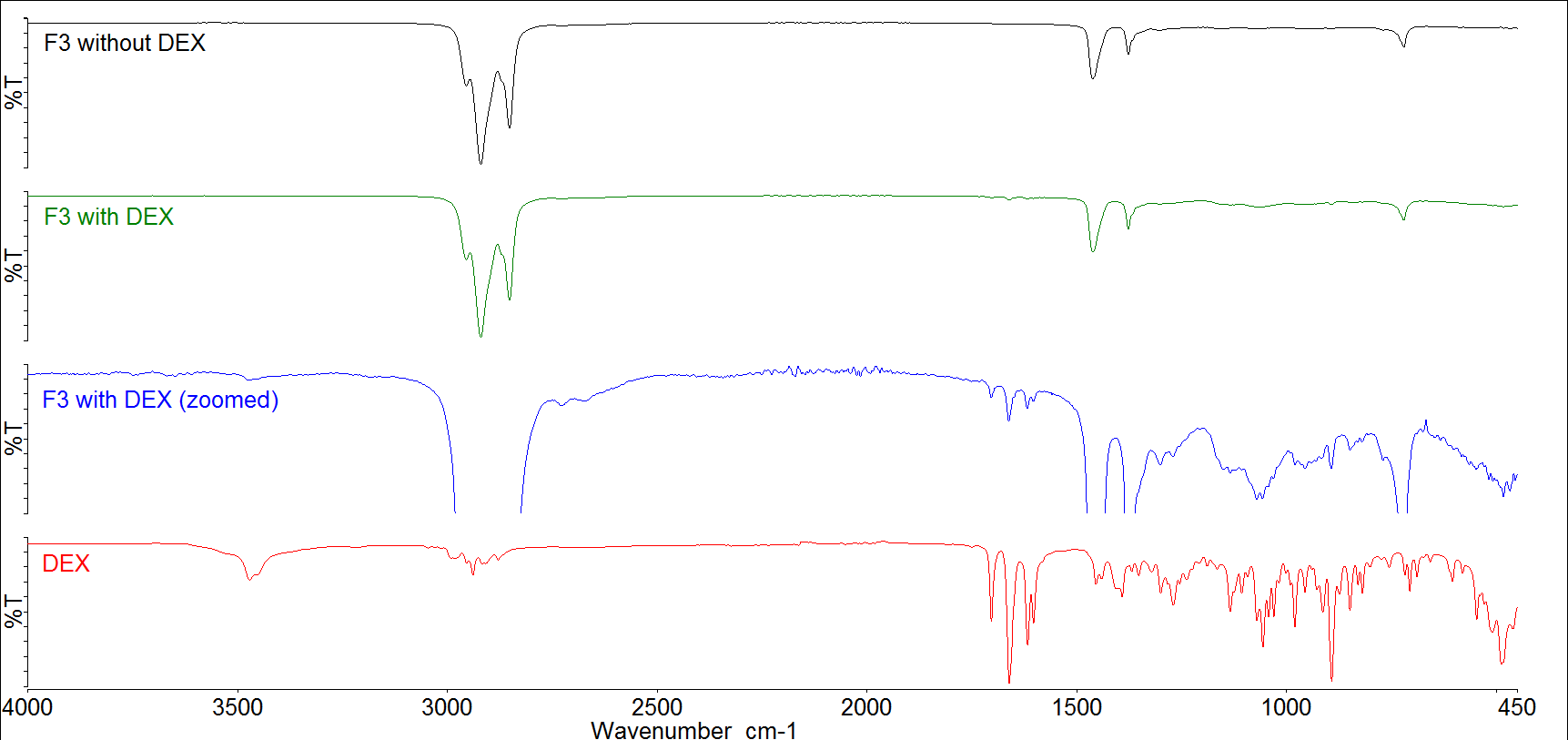

Supplement: Supplementary file 1 [file gels-11-00026-s001.zip › Figure S7.png]
